# Variability of a Non‐Centrosymmetric Tecto‐Borosulfate: Introduction of Various Molecular Cations into BiX[B(SO_4_)_2_]_4_ (X  =  H_3_O^+^, NO_2_^+^, NH_4_^+^, NO^+^)

**DOI:** 10.1002/chem.202502439

**Published:** 2025-10-05

**Authors:** Erich Turgunbajew, Matthias Hämmer, Lkhamsuren Bayarjargal, Florian Pielnhofer, Henning A. Höppe

**Affiliations:** ^1^ Lehrstuhl für Festkörperchemie Universität Augsburg Universitätsstr. 1 86159 Augsburg Germany; ^2^ Institut für Geowissenschaften Universität Frankfurt Altenhöferalle 1 60438 Frankfurt Germany; ^3^ Institut für Anorganische Chemie Universität Regensburg Universitätsstr. 31 93053 Regensburg Germany

**Keywords:** borosulfates, DFT, frameworks, NLO materials, silicate‐analogous materials

## Abstract

Although the number of borosulfates increased strongly over the past years, structures comprising a 3D anionic framework are still scarce. Herein, with BiX[B(SO_4_)_2_]_4_ (X  =  NH_4_^+^, NO_2_^+^, NO^+^) we present three new representatives. The bismuth borosulfates were synthesized under solvothermal condition and crystallize homeotypically to Bi(H_3_O)[B(SO_4_)_2_]_4_ in the tetragonal space group *I* $\bar{4}$ (Nr.82) (*a* = 11.827(6) – 11.908(1) Å, *c* = 8.1160(2) – 8.1315(6) Å, *Z* = 2). Via all vertices connected BS_4_ supertetrahedra reveal a zeolite type like structure like its found for the tectosilicate K_1.14_[Mg_0.57_Si_1.43_O_4_] emphasizing their close relationship. Underlining the weak coordination behavior, for the first‐time nitrosonium and nitrosylium cations are introduced into borosulfate chemistry. SHG measurements confirm the absence of an inversion center and show a nonlinear optical response with intensities up to 1.4 times higher than KDP. Furthermore, the titled compounds were characterized thoroughly by both single‐crystal and powder X‐ray diffraction, infrared spectroscopy, density functional theory (DFT) calculations, and thermal analysis.

## Graphical abstract

To date, Bi(H_3_O)[B(SO)_2_]_4_ is only the second structure type containing a 3D anion within borosulfate chemistry. In this contribution three more representatives were synthesized, formally replacing oxonium by the molecular cations nitrosonium, nitronium, and ammonium. The structures crystallize homeotypically to the very first compound. A careful adjustment of the educts even allows mixed representatives.

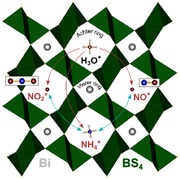

## 1 Introduction

More than a decade has passed since the first borosulfate K_5_[B(SO_4_)_4_] was reported.^[^ ^1^ ^]^ Since then, the number of known compounds has been steadily increasing, with over a hundred borosulfates up to now.^[^ ^2^ ^]^ The history of borosulfates shows rising interest in this comparably new material class. Like with any new material class the exploration first focuses on the numerical expansion of known compounds, using a variety of different elements as well as the fundamental research in which the basic chemistry is studied and tried to be understood. Even though this field of research is comparably new and still the named exploration occupies a large part of the work, over the time structure property relationships came to the fore which revealed great properties and opened first possible application fields. Ranging from host structures for luminescent materials,^[^ ^3^ ^]^ NLO active materials for lasers,^[^ ^4^, ^5^, ^6^ ^]^ proton conduction^[^ ^7^ ^]^ for solid state batteries and even catalysis^[^ ^8^ ^]^ may be possible in the future.

This versatility is based on the variability of this material class. Conventional borosulfates are defined as structures which are build up by alternating SO_4_ and BO_4_ tetrahedra sharing common corners. They feature a common basic building unit, the so‐called B(SO_4_)_4_^5−^ supertetrahedra. Based on the condensation degree noncondensed structures as in K_5_[B(SO_4_)_4_],^[^ ^1^ ^]^ ring structures like in the rare earth compounds R_2_[B_2_(SO_4_)_6_],^[^ ^3^ ^]^ chain‐like structures as in (NH_4_)_3_[B(SO_4_)_3_] or Mg[B_2_(SO_4_)_4_],^[^ ^9^, ^10^ ^]^ layered structures as in α‐Cu[B_2_(SO_4_)_4_]^[^ ^11^ ^]^ or even 3D networks appearing in Li[B(SO_4_)_2_^[^ ^12^ ^]^ and Bi(H_3_O)[B(SO_4_)_2_]_4_^[^ ^13^ ^]^ can be formed. Due to their analogy to silicates, for borosulfates the same nomenclature is employed, hence, they are classified as neso‐, ino‐, phyllo‐, and tectosilicate‐analogues.^[^ ^14^ ^]^ In addition to conventional, also unconventional borosulfates with S─O─S and B─O─B bridges as in Cs[B(SO_4_)(S_2_O_7_)]^[^ ^15^ ^]^ and Pb[B_2_O(SO_4_)_6_]^[^ ^16^ ^]^ are known violating Loewenstein´s^[^ ^17^ ^]^ and Pauling´s fourth rule,^[^ ^18^ ^]^ respectively. Recently, also planar BO_3_ units in X_2_[B_4_O_2_(SO_4_)_2_] (X = NH_4_^+^, K^+^, Rb^+^, Cs^+^) were introduced to borosulfates^[^ ^6^, ^19^ ^]^ otherwise only known in borate^[^ ^20^ ^]^ and borophosphate^[^ ^21^ ^]^ chemistry so far, underlining their variability and great potential. Evaluating the distribution of borosulfates a clear tendency toward lower condensation degrees is evident. Up to now over forty neso‐ while only two tectosilicate analogue compounds are known, namely Li[B(SO_4_)_2_]^[^ ^12^ ^]^ and Bi(H_3_O)[B(SO_4_)_2_]_4_^[^ ^13^ ^]^ are known.^[^ ^2^ ^]^ Only recently, has this number slightly increased with the discovery of the modular system M^III^M^I^[B(SO_4_)_2_]_4_ (M^III^ = Bi^3+^, Sb^3+^, Lu^3+^; M^I^ = H_3_O^+^, NO_2_^+^, Li^+^, Na^+^, K^+^, Rb^+^, Cs^+^) comprising the same anion topology as in Bi(H_3_O)[B(SO_4_)_2_]_4_.^[^ ^22^ ^]^ Apart from the quaternary system of BaO─B_2_O_3_─SO_3_ where the connection patterns can be directed from S─O─S over B─O─S to B─O─B bridges by varying the initial SO_3_ content together with temperature and time, no such adjustments to control the dimensionality are known so far. Additionally, the restricted number of known tectosilicate‐analogous compounds makes it difficult to recognize a pattern which parameters are relevant for the formation of these.

Regarding the cations that are found in borosulfates to achieve charge compensation a clear trend toward the most basic alkaline and earth alkaline elements is evident being accountable for around half of all known borosulfates. The remaining compounds are distributed to molecular NH_4_^+^ and H_3_O^+^ cations, d‐row, main group, and rare earth elements. Here, the main group elements have the smallest contribution with only four compounds known to date.^[^ ^13^, ^16^, ^23^ ^]^

Therefore, the titled compounds hold a special position. BiX[B(SO_4_)_2_]_4_ (X  =  H_3_O^+^, NH_4_^+^, NO_2_^+^, NO^+^) not only increase the number of tectosilicate‐analogues but also for the first time introduce nitronium and nitrosonium to borosulfates, so far only known for borate^[^ ^24^, ^25^ ^]^ and sulfate^[^ ^26^, ^27^, ^28^ ^]^ chemistry. Furthermore, they feature two differently charged cations, a hitherto unique feature in borosulfate chemistry, which is only known for the modular system M^III^M^I^[B(SO_4_)_2_]_4_ (M^III^ = Bi^3+^, Sb^3+^, Lu^3+^; M^I^ = H_3_O^+^, NO_2_^+^, Li^+^, Na^+^, K^+^, Rb^+^, Cs^+^) to which these compounds belong. Single crystal X‐ray diffraction (SCXRD) measurements not only reveal the existence of pure compounds with one monovalent cation species but also mixed structures, for example, Bi(NH_4_)*_x_* (NO_2_)_1−_ *_x_* [B(SO_4_)_2_]_4_ with controllable ratios of ammonium to nitronium. These results are confirmed by vibrational spectroscopy. Furthermore, vibrational spectroscopy studies focus on the position and chemical nature of the monovalent cation influencing the vibrational spectra. The findings are fostered by calculated IR active modes. Additionally, we elucidate the crystal structure, characterize the compounds by powder X‐ray diffraction (PXRD), second harmonic generation (SHG) measurements, and study the thermal decomposition process by thermogravimetric analysis (TGA) as well as temperature programmed powder X‐ray powder diffraction (TPPXRD).

## 2 Results and Discussion

### 2.1 Crystal Structure

**Figure 1:**
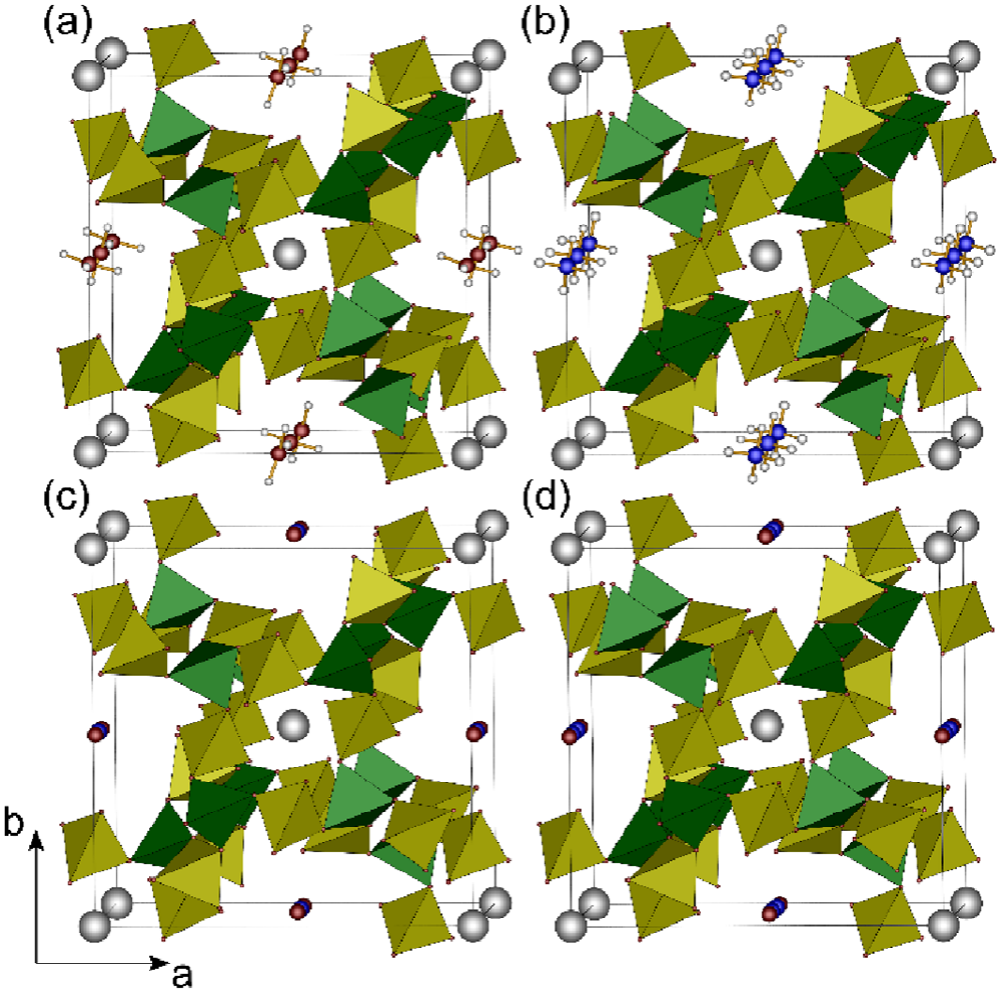
Unit cells of BiX[B(SO4)2]4 (X  = a) H_3_O^+^, b) NH_4_^+^, c) NO^+^, d) NO_2_^+^); sulfate tetrahedra are depicted in yellow, borate tetrahedra in green and bismuth atoms in grey.

**Figure 2:**
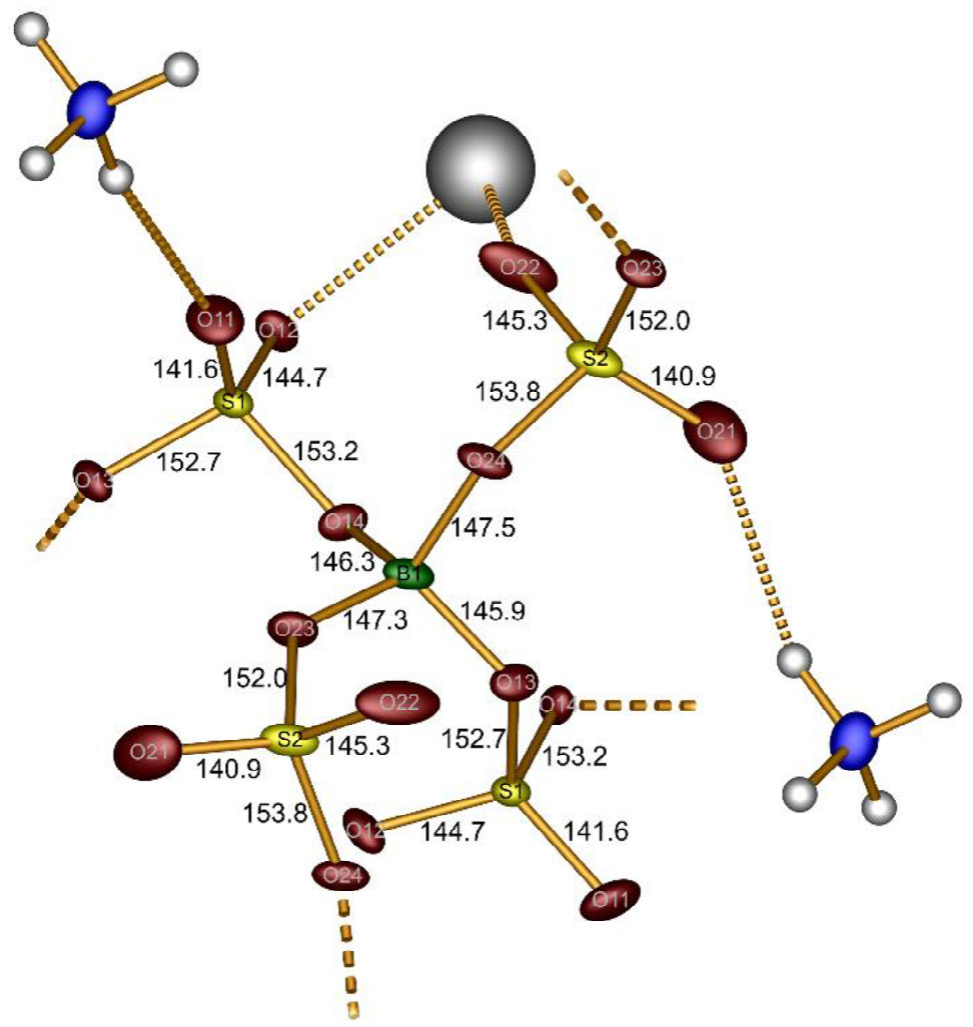
B(SO_4_)_4_^5−^ unit with the corresponding atom labelling, bond distances (in pm) and coordinated ammonium and bismuth cations exemplified once for each sulfate species. Dotted lines depict the connection to adjacent building units. Thermal ellipsoids are set to 70% probability.

**Figure 3:**
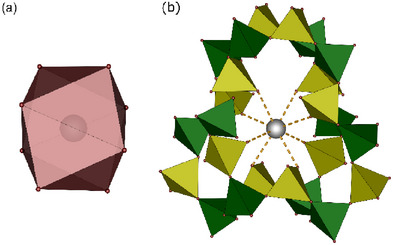
a) distorted square antiprismatic coordination polyhedron and b) extended coordination environment of Bi^3+^ in Bi(NO)[(SO_4_)_2_]_4_.

The borosulfates BiX[B(SO_4_)_2_]_4_ (X = H_3_O^+^, NH_4_^+^, NO_2_^+^, NH_4_^+^/NO_2_^+^, NO^+^) crystallize in the tetragonal space group *I* $\bar{4}$ homeotypic to Bi(H_3_O)[B(SO_4_)_2_]_4_ (Figure 1).^[^ ^13^ ^]^ They all comprise the same anion topology: B(SO_4_)_4_^5−^ supertetrahedra are condensed via all four sulfate moieties to further building units resulting in a 3D network with a ratio *T*:*X* = 1:2 (T  =  B, *X*  =  SO_4_) (Figure 2). With deviations below 1% all tetrahedra can be considered as regular (Table S12).^[^ ^29^, ^30^ ^]^ Due to the stark structural resemblance to silicates, the nomenclature introduced by Liebau is feasible.^[^ ^14^ ^]^ Accordingly, viewing along [001] layers comprising *vierer* and *achter* rings are observed. The same topology is also known for the tectosilicate K_1.14_[Mg0_.57_Si_1.43_O_4_] comprising also edge‐sharing *vierer* and *achter* rings formed by the anion (Figure S1). Considering the third dimension, channels along [001] are formed which host the differently charged cations. Bismuth, located in the channels of the *vierer* rings, lies on a 2*a* site with $\bar{4}\;$ symmetry, surrounded square antiprismatically by eight oxygen atoms (Figure 3). This quite symmetrical environment denotes no influence of the lone pair. The qualitative influence of the lone pair is accessible via several routes, for example, ELF calculations, the Born effective charge Z* or by simple geometrical calculations. For the latter, we employ a rather new approach using the centroid deviation of the cation within the coordination polyhedron which provides comparably fast and easily accessible results from single crystal data.^[^ ^29^, ^30^, ^31^ ^]^ As the centroid deviation is nearly zero here, the lone pair can be safely regarded as sterically inactive (Table S11). Another indication which underlines this presumption are the interatomic distances being in good agreement with the sums of the ionic radii (Table S13). Only considering the bismuth cations, a distorted bcc substructure is apparent.

The monovalent cations are situated in the channels generated by the *achter* rings, occupying different or even multiple sites. Out of the introduced cations only nitronium occupies a 2*d* site with $\bar{4}$ symmetry and shows no disorder. Similarly to the bismuth substructure, the NO_2_^+^ ions build up a distorted bcc structure shifted by the vector [½, 0, ¼]. Furthermore, the interaction with the anion can be considered ionic. The other three oligoatomic cations H_3_O^+^, NH_4_^+^, and NO^+^ occupy 4*f* sites with site symmetry 2 and show disorder with an occupation of 50 %. While the interaction of NO^+^ again can be considered as ionic, H_3_O^+^ and NH_4_^+^ generate hydrogen bonds to terminal oxygen atoms of the polyanion. The oxonium cation realises medium strong hydrogen bonds to two terminal oxygen atoms classified by donor acceptor distances of 278 pm (Figure S2). Likewise, ammonia is bound via two medium strong hydrogen bonds, additionally to two weak ones with donor acceptor distances of 297 and 329 pm (Figure S3).^[^ ^32^ ^]^ In addition to these pure compounds with only one type of monovalent cation embedded, also mixed variations namely Bi(NH_4_)*_x_* (NO_2_)_1−_ *_x_* [B(SO_4_)_2_]_4_ with varying parameter *x* were synthesized (for an example see Table S1 in the supplement). Here, the cations occupy the same positions like in the respective pure compounds. In this system, it appears that the ratio is controllable by the variation of the respective educt ratio with having a higher tendency to include ammonia into the structure. Starting with stoichiometrically equal amounts of nitrate in Bi(NO_3_)·5H_2_O and ammonia in NH_4_(SO_4_)_2_ results in about 63% occupation of ammonia in the structure. These results demonstrate an average value obtained by SC‐XRD measurements of several single crystals and are confirmed by vibrational spectroscopy which are discussed in the following section.

### 2.2 Infrared Spectroscopy

Vibrational spectroscopy was performed which proved useful to differentiate the structurally similar compounds. In the PXRD patterns (Figure S4) which exhibit only slight changes in the respective reflection intensities and positions due to somewhat different cell parameters, the monovalent cations show a comparably small influence contrary to the vibrational spectroscopy. Here, the element‐specific vibrational bands of the monovalent cations identify the compounds unambiguously. Furthermore, the cation influence in the fingerprint region of the anion discloses their position and crystal chemical surrounding. All recorded data were compared to simulated spectra obtained by DFT modelling.

**Figure 4:**
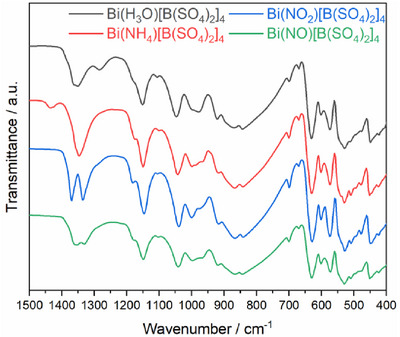
Infrared spectra of BiX[B(SO4)2]4 (X = H_3_O^+^ (grey), NH_4_^+^ (red), NO_2_^+^ (blue), NO^+^ (green) in range between 1500‐ 400 cm^−1^.

**Figure 5:**
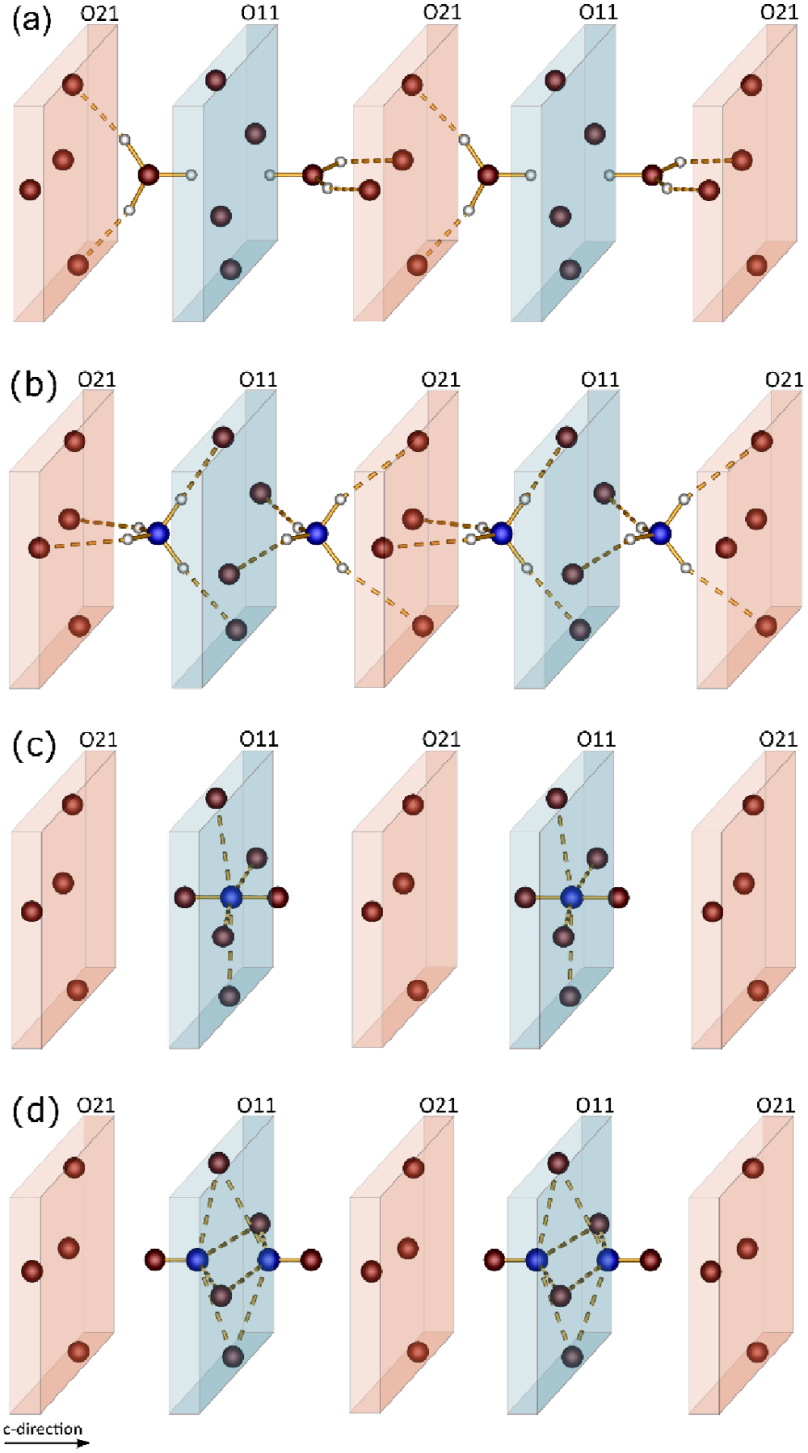
Position and coordination of a) oxonium, b) ammonium, c) nitronium and d) nitrosonium with planes belonging to different oxygen species O11 (blue) and O21 (red).

**Figure 6:**
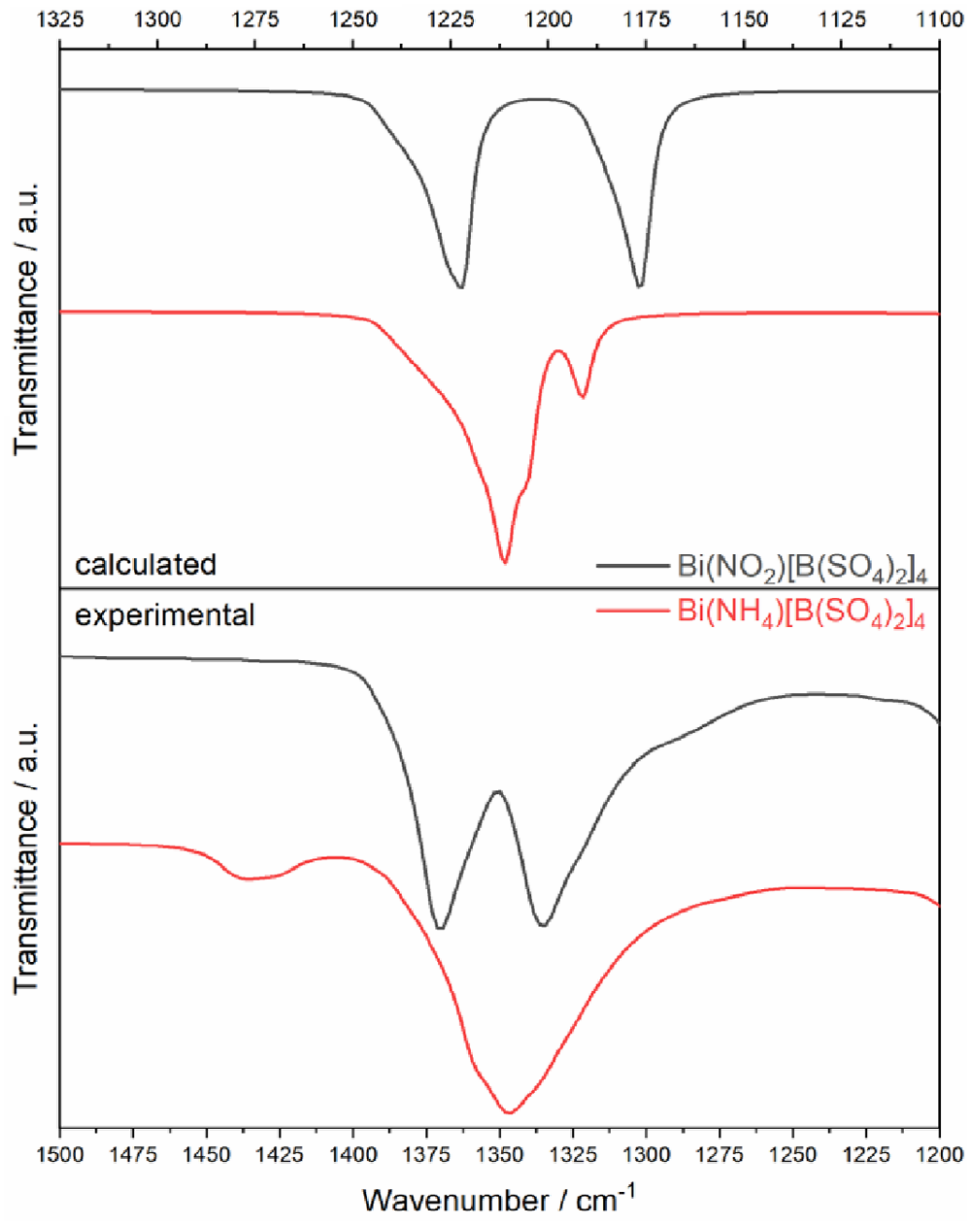
Magnified section of the calculated (upper) and experimental (lower) infrared spectra of BiX[B(SO_4_)_2_]_4_ (X = NH_4_ (red), NO_2_ (grey)) in the respective region for terminal S‐O stretching vibrations.

Accordingly, ammonium exhibits an asymmetric stretching mode *ν*_as_(N─H) at 3282 cm^−1^ (DFT: 3200 – 3352 cm^−1^) in addition to an asymmetric deformation mode *δ*_as_(N─H) around 1435 cm^−1^ (DFT: 1432 – 1449 cm^−1^). Likewise, nitronium can be identified by its asymmetric stretching mode *ν*_as_(O─N─O) peaking at 2368 cm^−1^ (DFT: 2309 cm^−1^). The stretching mode *ν*(N─O) of nitrosonium appears around 2273 cm^−1^ (DFT: 2098 cm^−1^) only as a very weak band. To validate the integration of NO^+^ into the framework, an additional Raman spectrum was recorded confirming the presence with a band located around 2300 cm^−1^ (Figure S5). Overall, all values are in good agreement with literature.^[^ ^33^, ^34^, ^35^ ^]^ For Bi(H_3_O)[B(SO_4_)_2_]_4_, the O─H modes of oxonium appear as broad bands situated around 2800 cm^−1^ and 1700 cm^−1^ (DFT: highest intensities at 2797 cm^−1^ and 1634 cm^−1^), respectively (Figure S6). Additionally, due to the hygroscopic character of borosulfates the compounds partially hydrolyze on the surface leading to the formation of sulfuric acid. Thus, the reaction with ambient moisture increases the intensity of these bands.^[^ ^36^ ^]^ This hypothesis was also confirmed by an experiment in which Bi(NH_4_)[B(SO_4_)_2_]_4_ was exposed to air for varying periods of time. The results are depicted in Figure S7 in the Supporting Information and clearly show a progressive increase in intensity of the named bands over time. The modes of the anion can be observed at lower wavenumbers, in the range of 1500 – 400 cm^−1^ (Figure 4) (full spectrum in Figure S6). At first sight, the spectra appear to be identical, which would be consistent with the homeotypic nature of the crystal structures. However, a closer look reveals slight deviations between recorded spectra and the DFT calculation; the deviation to the experimental values is caused by a systematic overestimation of the bond lengths in the anionic borosulfate network causing a larger cell volume, which is typical for GGA‐ based functionals. As has been demonstrated by several theoretical studies on borosulfates, asymmetric stretching modes *ν*_as_(S─O_term_) are typically found in the region around 1350 cm^−1^ (DFT: around 1200 cm^−1^).^[^ ^37^, ^38^, ^39^ ^]^ Depending on the crystal chemical surrounding of the monovalent cation present, characteristic patterns are observed. Generally, it is well known that intermolecular interaction of a molecule can cause symmetry reduction. This then yields frequency shifts and possible splitting of degenerate modes or even activation of otherwise inactive IR or Raman vibrations.^[^ ^40^ ^]^ Transferring this idea to a free sulfate tetrahedron a study by Nakamoto et al^[^ ^41^ ^]^ revealed a symmetry reduction from *T*_d_ to *C*_2v_ in Co(III) sulfato complexes which was caused by monodentate, bidentate, or bridging bidentate coordination to the cobalt cation. This reduction led to a splitting of the antisymmetric stretching mode *ν*_3_ similar to our findings. An alternative approach to evaluating this observation may be to consider the position of the monovalent cation in the channels and, consequently, the interaction with their direct coordination environment. The position of the latter can be evaluated by considering two oxygen species namely O11 and O12 forming alternating layers within the channel (Figure 5). Accordingly, the cations either occupy a position within or in between leading to either one or two bands, respectively. Ammonium shows disorder with an occupation of 50% for both positions; therefore, in average it is coordinated to all terminal oxygen atoms equally resulting in only one band. This was modelled by DFT by creating two fully ordered structures. The simulated IR spectra of both models are very similar (Figure S8) and show a small splitting of the terminal S─O vibrations at 1210 cm^−1^, 1204 cm^−1^, and 1191 cm^−1^ (model 2: 1187 cm^−1^). In contrast, nitronium is situated within a plane and accordingly is coordinated to a single oxygen species resulting in two bands in which the coordinated ions are found at higher and noncoordinated at lower wavelengths. This is resembled by the simulated spectrum with a splitting of the two most intense signals at 1221 cm^−1^ and 1175 cm^−1^. Nitronium therefore leads to a much larger splitting (Δ = 46 cm^−1^) than ammonium (Δ = 19 cm^−1^) which is in good agreement with the experimental observation and supports the explanation of the coordination environment of the cation being responsible for the large split of the IR signal (Figure 6). The largest splitting is observed for H_3_O^+^ with a splitting of 100 cm^−1^, also supported by DFT calculations for the two fully ordered models.

Considering the remaining regimes, the spectra coincide very well. Hence, asymmetric and symmetric stretching modes *ν*_s/as_(B─O) occur in the region between 1180 cm^−1^ and 990 cm^−1^, followed by symmetric stretching modes *ν*_s_(S─O) between 920 cm^−1^ and 830 cm^−1^. Below, starting from 700 cm^−1^, solely bending vibrations *δ*(O─S─O, O─B─O, S─O─B) can be found. For a more detailed assignment, we refer to Tables S15 – S19 in the Supporting Information.

To demonstrate the tunability of the ratios between different kinds of monovalent cations, beside the pure compounds, a series of mixed variations with different ratios of ammonium to nitronium was synthesized (Figure S9). From top to bottom, a continuous increase in intensity of the nitronium mode around 2372 cm^−1^ is consistent with a corresponding decrease in intensity of the ammonium modes around 3280 cm^−1^ and 1430 cm^−1^. Following the evolution of the asymmetric stretching modes *ν*_as_ (S─O_term_) around 1350 cm^−1^, as expected, analogously with increasing nitronium content a continuous transition from a single, to two separate bands is evident with a crossover area where both spectral contributions sum up independently. The results coincide qualitatively well with the findings of the SC‐XRD measurements. Furthermore, based on this series of nitronium ammonium bismuth borosulfates we assume that the insertion of different monovalent cations with tunable ratios constitutes a universal rule that is also true for the whole structural family.^[^ ^22^ ^]^

### 2.3 Nonlinear Optical Properties

**Figure 7:**
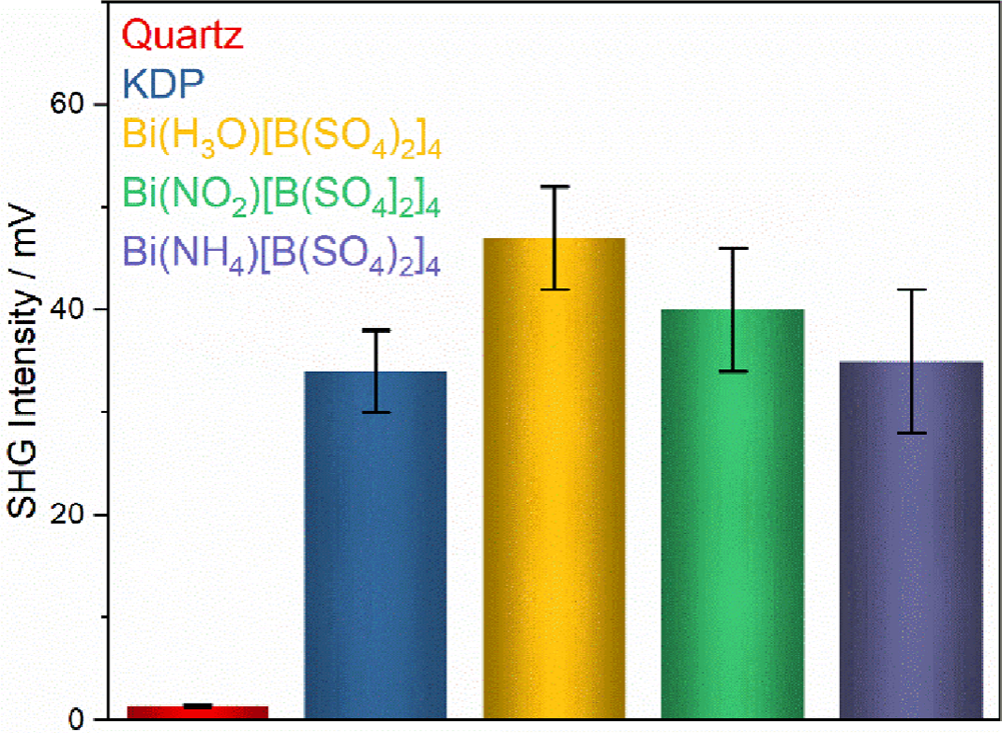
SHG intensities of BiX[B(SO_4_)_2_]_4_ (X = NH_4_^+^, H_3_O^+^, NO_2_^+^) compared to the reference materials KDP and quartz (grain size < 25 µm).

SHG measurements were performed for BiX[B(SO_4_)_2_]_4_ (X = H_3_O^+^, NH_4_^+^, NO_2_^+^, NO^+^) with two different grain sizes and reference samples using the powder method of Kurtz and Perry.^[^ ^42^, ^43^ ^]^ All samples show very strong SHG signals in comparison to reference centrosymmetric materials such as corundum (Al_2_O_3_) or an empty glass capillary which generates no SHG‐signals (0 mV). The higher than zero SHG‐intensity of our samples confirms the absence of an inversion center, as well as the results of the crystal structure determination by SC‐XRD measurements. In addition, the measured intensities are much higher than quartz and comparable to those of the reference material potassium hydrogen phosphate (KDP), even reaching up to 1.2–1.4 times higher (Figures 7 and S10).^[^ ^44^ ^]^ In contrast to a previous publication, where we only reported the SHG intensity of Bi(H_3_O)[B(SO_4_)_2_]_4_ without grain sizes,^[^ ^13^ ^]^ we have provided here SHG measurements considering different grain sizes. The high SHG intensities of samples can be caused by either high SHG coefficients or phase‐matching conditions like in KDP. SHG measurements with many different crystallite sizes would allow us to distinguish between matchable and non‐phase‐matchable materials. Unfortunately, measurements with two different grain sizes are not sufficient to reliably determine phase‐matching conditions. However, measurements with the same grain sizes and under the same experimental conditions help to understand the origin of SHG effects. The comparable high SHG signals of other homeotypic borosulfates indicate possible high nonlinear effects of individual building units and higher SHG coefficients. Generally, the total nonlinear optical response mainly has its origin in the anionic part of the compounds as proposed by the anionic group theory. However, an influence caused by the different monovalent cations seems evident as all compounds contain the same 3D anion. The cationic impact on SHG intensities usually occurs only for specifically polarizable atoms and building units.^[^ ^45^, ^46^ ^]^ For instance, asymmetric building units generated by a second‐order‐Jahn–Teller effect, polar displacements by d^10^ elements, large and soft cations with a high polarizability as well as structural distortions caused by atoms with stereochemically active lone pairs should be mentioned.^[^ ^47^, ^48^, ^49^, ^50^ ^]^ We observed a decrease of SHG signals for different molecular building units from the oxonium to the ammonium, and thus an influence of molecular cations. As the influence of the ions contributing to the SHG intensities is proportional to their polarizability the decrease from the oxonium via the nitronium to the ammonium compound can be well understood.^[^ ^51^, ^52^ ^]^

### 2.4 Thermal Analysis

The thermal decomposition processes of the titled compounds were investigated using TGA and TPPXRD. In accordance with their structural resemblance, the TGA shows similar behavior for all four compounds, suggesting the same decomposition process, regardless of the monovalent cation incorporated (Table S14; Figure S11). Consequently, the discussion will be limited to Bi(NO)[B(SO_4_)_2_]_4_ only. Following the release of adhesive sulfuric acid at approximately 180 °C, as confirmed by PXRD measurements (Figure S12), the compounds begin to decompose at around 250 °C to form Bi_2_(SO_4_)_3_ and B_2_O_3_ according to this reaction equation:

(1) $$\begin{array}{cc} 2Bi\left(\mathrm{NO}\right){\left[B{\left(SO_{4}\right)}_{2}\right]}_{4} & \to Bi_{2}{\left(SO_{4}\right)}_{3}+4B_{2}O_{3}+2\mathrm{NO}\left(g\right)\\ & \;+\;13SO_{3}\left(g\right)+\frac{1}{2}O_{2}\left(g\right) \end{array}$$

**Figure 8:**
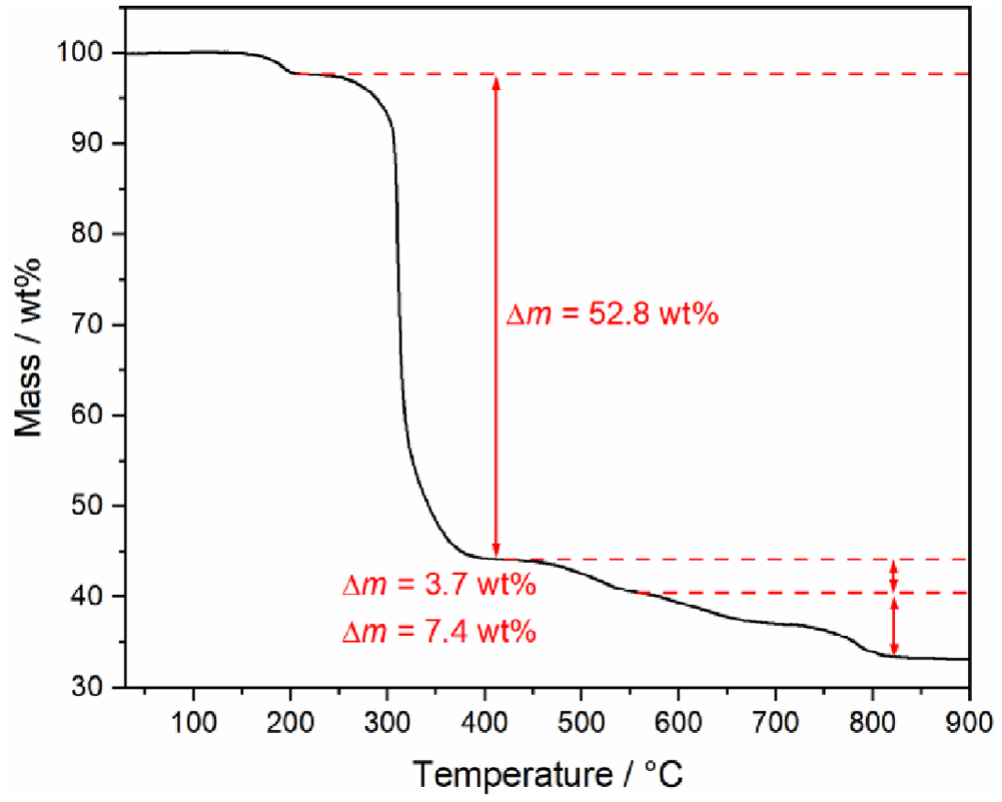
Thermogravimetric analysis of Bi(NO)[B(SO_4_)_2_]_4_ under nitrogen atmosphere.

**Figure 9:**
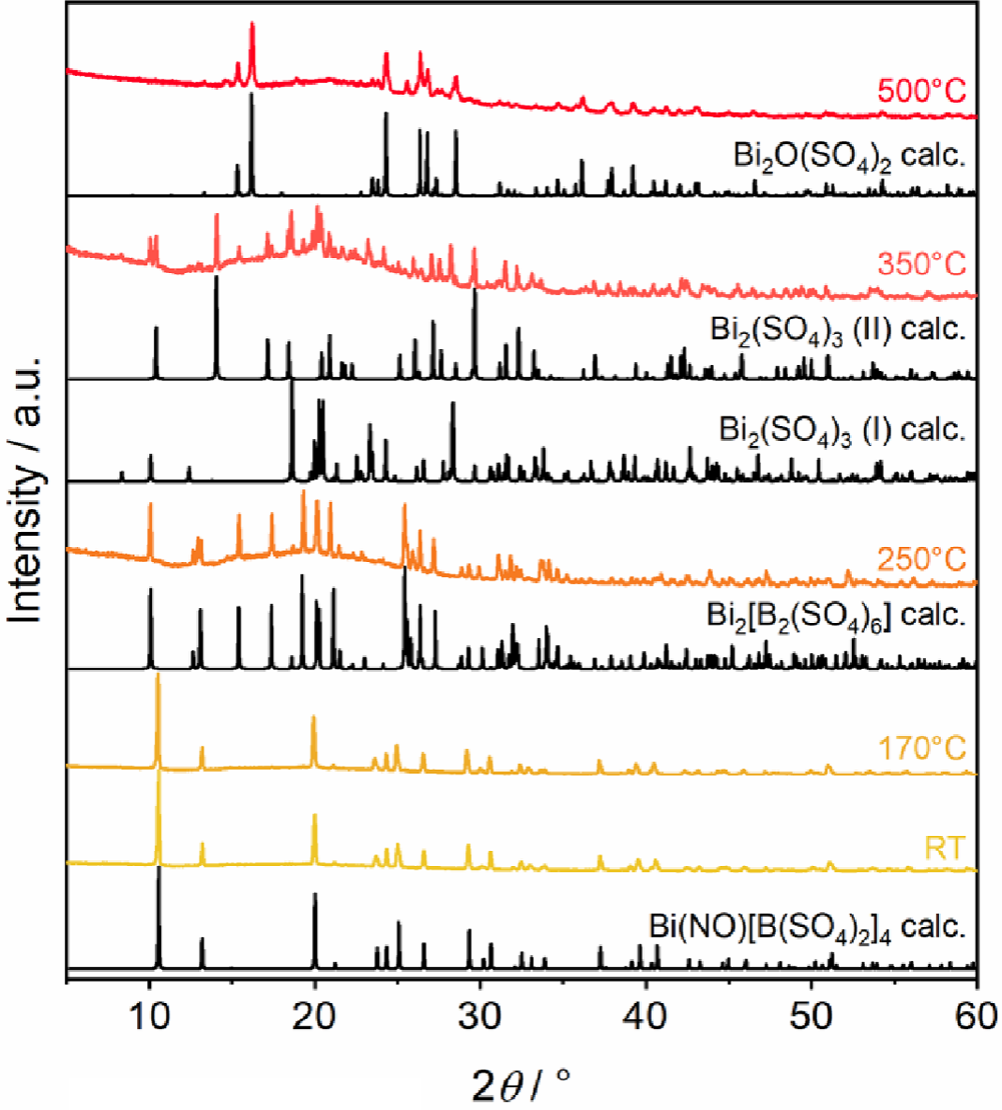
TPPXRD patterns of Bi(NO)[B(SO_4_)_2_]_4_ between room temperature and 500 °C in comparison to simulated patterns of Bi(NO)[B(SO_4_)_2_]_4_, Bi_2_[B(SO_4_)_6_],^[^ ^11^ ^]^ two modifications of Bi_2_(SO_4_)_3_^[^ ^25^, ^37^ ^]^ and Bi_2_O(SO_4_)_2_.^[^ ^39^ ^]^

The observed mass loss of 52.8% agrees well with the calculated mass loss of 51.9% and corresponds to the release of 6.5 formular units SO_3_ (Figure 8). In contrast, the TPPXRD measurements confirm the formation of the intermediate Bi_2_[B_2_(SO_4_)_6_] that forms at 250 °C which subsequently decomposes to Bi_2_(SO_4_)_3_ at around 350 °C (Figure 9). This observation suggests an alternative decomposition pathway which can be formulated as follows:

(2) $$\begin{array}{cc} 2Bi\left(\mathrm{NO}\right){\left[B{\left(SO_{4}\right)}_{2}\right]}_{4} & \to Bi_{2}\left[B_{2}{\left(SO_{4}\right)}_{6}\right]+3B_{2}O_{3}\\ & \;+\;2\mathrm{NO}\left(g\right)+10SO_{3}+\frac{1}{2}O_{2}\left(g\right) \end{array}$$

(3) $$Bi_{2}\left[B_{2}{\left(SO_{4}\right)}_{6}\right]\to Bi_{2}{\left(SO_{4}\right)}_{3}+B_{2}O_{3\;}+3SO_{3}$$

We assume that this discrepancy presents a rare case where both methods do not correspond and arises from the intrinsic nature of each method. While the TGA experiment is conducted in an open system, whereby the emerging volatile gases can be readily eliminated, the decomposition process in the TPPXRD measurement occurs in a closed system inside a capillary. In conjunction with the evaporating adhesive sulfuric acid, the released five formula units SO_3_ generate an environment which favors the formation of the intermediate Bi_2_[B_2_(SO_4_)_6_] over the immediate decomposition product Bi_2_(SO_4_)_3_.^[^ ^31^, ^53^ ^]^ This hypothesis of gas evolution is supported by the fact that the capillaries exploded during the TPPXRD experiments. A comparable observation was already made for Sr[B_2_(SO_4_)_4_].^[^ ^9^ ^]^ The TGA suggested a one‐step decomposition toward Sr(SO_4_) around 400 °C. Alternatively, a thermal treatment in the mother liquor at 300 °C yielded a transformation toward Sr[B_2_O(SO_4_)_3_]. The results obtained from TPPXRD measurements of Sr[B_2_(SO_4_)_3_(S_2_O_7_)] further corroborate the results as they also confirm the presence of Sr[B_2_O(SO_4_)_3_] as an intermediate phase.^[^ ^54^ ^]^ It seems that during the decomposition, the atmosphere has a significant influence on the decomposition of borosulfates and such transformations can already occur at low amounts of additional sulfuric acid or SO_3_ as shown by the latter case. Subsequently, both methods are in accordance. Bi_2_(SO_4_)_3_ decomposes around 500 °C toward Bi_2_O(SO_4_)_2_ (eq. 4) which corresponds well to the observed mass loss of 3.7% (Δ*m*_calc_ = 3.7%) and was also proven by TPPXRD measurements (Figure 9). As proposed by *Aurivillius* et al,^[^ ^55^, ^56^ ^]^ Bi_2_O(SO_4_) presumably decomposes via several intermediates like Bi_26_O_27_(SO_4_)_12_ and Bi_14_O_16_(SO_4_)_5_ to form Bi_2_O_3_ and B_2_O_3_ which is in accordance with the observed mass loss of 7.5% (Δ*m*_calc_ = 7.4%) (eq. 5). Due to the loss of crystallinity, the final product could not be characterized.

(4) $$Bi_{2}{\left(SO_{4}\right)}_{3}\to Bi_{2}O{\left(SO_{4}\right)}_{2}+SO_{3}\left(g\right)$$

(5) $$Bi_{2}O{\left(SO_{4}\right)}_{2}\to Bi_{2}O_{3}+2SO_{3}\left(g\right)$$

The thermal stabilities of BiX[B(SO_4_)_2_]_4_ (X = H_3_O^+^, NH_4_^+^, NO_2_^+^, NO^+^) confirm the general trends that were observed for borosulfates so far, where compounds with decreasing condensation degrees are typically increasingly stable.^[^ ^3^ ^]^ A comparison of compounds comprising solely B─O─S bridges was made in Table 1 and demonstrates a direct correlation between the dimensionality and their thermal stabilities. Accordingly, the titled compounds comprise the lowest thermal stability so far.

**Table 1:** Thermal stabilities of reported borosulfates comprising solely B─O─S bridges.

Compound	*T*_decomp_ [°C]	Dimensionality	Refs.
Eu_2_[B_2_(SO_4_)_6_]	570	0 D	[3]
Rb_5_[B(SO_4_)_4_]	530	0 D	[57]
Na_5_[B(SO_4_)_4_]	434	0 D	[12]
K_3_[B(SO_4_)_3_]	396	1 D	[12]
Sr[B_2_(SO_4_)_4_]	400	1 D	[9]
Cd[B_2_(SO_4_)_4_]	330	2 D	[58]
Bi(NO)[B(SO_4_)_2_]_4_	250	3 D	this work

## 3 Conclusions

In this contribution, we presented the crystal structures of the bismuth borosulfates Bi*X*[B(SO_4_)_2_]_4_ (*X*  =  NH_4_^+^, NO_2_^+^, NO^+^) which are homeotypic with the second known 3D borosulfate. The structures consist of supertetrahedra connected via all four corners creating a 3D structure which shows similarities to the zeolite K_1.14_[Mg_0.57_Si_1.43_O_4_] and emphasizes the analogy to silicates. For the first time, nitronium and nitrosonium were introduced into borosulfate chemistry a phenomenon known only for weakly coordinating structures. Due to the crystallization in a non‐centrosymmetric space group, we performed SHG measurements and confirmed the structure refinement. Additionally, SHG intensities with up to a factor of 1.4 higher compared to the reference material of KDP were measured.

IR spectroscopy proved to be a highly effective method discriminating very similar compounds. It was possible to differentiate between the compounds not only based on the characteristic vibrations of their molecular cations, but also on terminal S─O_term_ vibrations in the fingerprint region. The position and interaction with the anionic framework led to specific shifts and splittings of the respective bands, which are characteristic for each cation. These findings were qualitatively confirmed by DFT calculations and simulated infrared spectra.

The thermal analysis revealed unusual discrepancies between the initial decomposition product in a TGA and TPPXRD experiments. We suggest that this discrepancy is due to the intrinsic differences between open and closed reaction conditions. In contrast to the open system (TGA), where volatile gases can evaporate immediately, in closed systems these gases are maintained but typically do not affect the decomposition processes and coincide well with the results of TGA experiments. However, in this case, comparably high amounts of SO_3_ dissipate during the first decomposition step apparently leading to a higher pressure and a reaction environment which preferably yields Bi_2_[B_2_(SO_4_)_6_] instead of the respective binary sulfate. A similar behaviour was found for Sr[B_2_(SO_4_)_4_] where a thermal treatment in the mother liquor yielded a transformation to Sr[B_2_O(SO_4_)_3_] which was not observed in the TGA experiment.^[^ ^9^ ^]^ The assumption of a strong gas evolution is corroborated by TPPXRD measurements in which the silica glass capillaries exploded.

The titled compounds revealed a high variability of molecular cations on the monovalent cationic site which gives rise to further investigation on alkali metals comprising similar ionic radii. Further investigations on substituting the trivalent cations by rare‐earth or antimony are in train and will be presented elsewhere.

## 4 Experimental Section

**Synthetic Procedure**: For all four compounds, the starting materials were ground in an agate mortar and transferred into silica ampules (length: 12 cm, diameter: 1 cm, wall thickness: 0.1 cm). Followed by a storage in the compartment drier for two minutes at 180 °C, oleum was added. With regard to Bi(NO)[B(SO_4_)_2_]_4_, prior to the addition of oleum the NOHSO_4_ solution was added. Subsequently, the ampules were torch sealed applying the following temperature program: heating to 180 °C with 50 °C/h, holding the temperature for 24 hours and cooling down with a rate of 6 °C/h to room temperature. In all cases, colorless crystals were obtained. Subsequently, the excess sulfuric acid was decanted. Prior to opening, the ampoules were frozen with liquid nitrogen. (**Caution**: During and even after the reaction, the ampules are under remarkable pressure and must therefore be handled with great care, for example, they must be cooled with liquid nitrogen before opening.) The moisture sensitive crystals were washed with dry acetonitrile (Acros, 99.9%, extra dry) and were subsequently transferred and stored in an argon filled glove box for further investigation. Phase purity was confirmed by PXRD (Figure S4). Supplementary crystallographic data are provided in reference.^[^ ^59^ ^]^

The precursor Bi_2_(CO_3_)O_2_ was synthesized hydrothermally according to Huang et al.^[^ ^60^ ^]^ Therefore, the educts Bi(NO_3_)_3_·5H_2_O (4 mmol, 1.94 g, Alfa Aesar 98 %) and urea (6.4 mmol, 0.38 g, Merck 99,5 %) were dissolved in 40 ml demineralized water, filled into PTFE pressure digestion vessels, 10 ml each, and kept in the compartment drier for 6 days at 200 °C. The product was washed several times with demineralized water and ethanol, (assisted by a centrifuge between each washing step (4000 rpm, 10 minutes)) and centrifugated with 4000 rpm for 10 minutes between each washing step. Finally, the colorless precipitate was dried in a compartment at 65 °C for 24 hours, realizing a yield of 96 %. Phase purity was confirmed by PXRD (Figure S6).

Bi(H_3_O)[B(SO_4_)_2_]_4_: Bi_2_(CO_3_)O_2_ (62,7 mg, 0.12 mmol), B(OH)_3_ (182.6 mg, 2.95 mmol, Merck > 99.5 %), Oleum 65 % SO_3_ (1 ml, Merck)

Bi(NH_4_)[B(SO_4_)_2_]_4_: Bi_2_O_3_ (56.1 mg, 0.12 mmol, Riedel‐de Haën, >99.5 %), B_2_O_3_ (100.5 mg, 1.4 mmol, Sigma‐Aldrich, >99 %), (NH_4_)_2_SO_4_ (94.1 mg, 0.7 mmol, Merck > 99.9 %), Oleum 65 % SO_3_ (1 ml, Merck)

Bi(NO_2_)[B(SO_4_)_2_]_4_: Bi(NO_3_)⋅5H_2_O (113.7 mg, 0.2 mmol, Alfa Aesar, >99.9 %), B_2_O_3_ (97.9 mg, 1.4 mmol, Sigma‐Aldrich, >99 %), Oleum 65 % SO_3_ (1 ml, Merck)

Bi(NO)[B(SO_4_)_2_]_4_: Bi_2_O_3_ (55.4 mg, 0.12 mmol, Riedel‐de Haën, >99.5 %), B_2_O_3_ (99.4 mg, 1.4 mmol, Sigma‐Aldrich, >99 %), NOHSO_4_ in sulfuric acid 40 wt % (0.2 ml, Sigma‐Aldrich), Oleum 65 % SO_3_ (1 ml, Merck)

**Single crystal X‐ray diffraction**: Immediately after opening the ampoule, single crystals were transferred from the mother liquor into perfluorinated polyether. Suitable crystals were picked under a polarization microscope, mounted on to a MicroMount (MiTeGen) and directly transferred into a cold nitrogen gas stream (Oxford Cryostream Plus). Single Crystal X‐ray Diffraction data were collected on a Bruker D8 Venture under Mo_Kα_ radiation (0.71 073 Å) equipped with a PHOTON‐100 detector. Absorption correction was performed via the multi scan method. The crystal structures were solved by direct methods and refined by full‐matrix least‐squares technique with the SHELXTL program.^[^ ^61^ ^]^ The hydrogen positions of ammonium within Bi(NH_4_)[B(SO_4_)_2_]_4_ were located using residual electron density and fixed to 1.03 Å.^[^ ^62^ ^]^ The N─O bond distance within the nitrosonium cation in Bi(NO)[B(SO_4_)_2_]_4_ was fixed to 1.04 Å.^[^ ^26^ ^]^ Further relevant crystallographic data is listed in the tables S1 – S9 in the Supporting Information.

**Powder X‐ray diffraction**: The samples were ground and filled into a Hilgenberg glass capillary (outer diameter 0.3 mm, wall thickness 0.01 mm). The PXRD patterns were recorded with a D8 Venture in transmission geometry using Cu‐Kα radiation (1.54 184 Å), a 1D LynxEye detector and a nickel filter to suppress *K*_β_ radiation. The TPPXRD patterns were recorded on the same device using a furnace attachment. The temperature was adjusted with a heating ramp of 0.5 °C/s and held for 30 minutes before each measurement. The strong background between 12.5°< 2*θ* <30° is due to the furnace attachment.

**Infrared Spectroscopy**: The infrared spectra were recorded using an EQUINOX 55 FT‐IR spectrometer (Bruker) equipped with a platinum ATR unit in the range of 4000 – 400 cm^−1^ and a resolution of 4 cm^−1^ with 32 scans per measurement.

**Second harmonic generation**: Second harmonic generation (SHG) measurements were performed on microcrystalline powder samples with grain sizes of < 25 µm and 25–50 µm in glass capillaries using the Kurtz–Perry approach. Corundum (Al_2_O_3_), glass capillary, quartz, and potassium dihydrogen phosphate (KDP, KH_2_PO_4_) were used as reference materials. A Q‐switched Nd:YAG laser (1064 nm, 5–6 ns, 2 kHz) was used for the generation of the fundamental pump wave. The fundamental infrared light was separated using a harmonic separator, a short‐pass filter, and an interference filter from the generated second harmonic (532 nm). The generated SHG signal was collected with a photomultiplier and an oscilloscope from five different areas of the sample. On each position, 128 pulses were measured and averaged. Background signals between the laser pulses were used to correct the measured intensities. The SHG measurements were performed under ambient conditions in transmission geometry.

**Thermogravimetric Analysis**: The thermogravimetric analyses were performed in alumina crucibles, under nitrogen atmosphere and a heating rate of 5 °C/min using a STA 409 PC Luxx.

**DFT calculations**: Quantum chemical calculations were performed in the framework of density functional theory (DFT) using a linear combination of Gaussian‐type functions (LCGTF) scheme as implemented in CRYSTAL23.^[^ ^63^, ^64^ ^]^ Full structural optimizations were carried with the GGA (PBE)^[^ ^65^ ^]^ xc‐functional. The energetical convergence criterion was set to 1 × 10^−8^ a.u. with a k‐mesh sampling of 6 × 6 × 6. All‐electron basis sets for B, S, O, N, and H were taken from refs. [66, 67, 68, 69, 70] and a pseudo potential for Bi ^[^ ^71^ ^]^ was used. The outermost coefficient for S was optimized to 0.515. IR spectra were simulated according to.^[^ ^72^ ^]^

## Supporting Information

The authors have cited additional references within the Supporting Information.^[^ ^73^, ^74^ ^]^

## Conflict of Interest

The authors declare no conflict of interest.

## Supporting information



Supporting Information

## Data Availability

The data that support the findings of this study are available from the corresponding author upon reasonable request.
