# Supplementary material for: Variability of a Non‐Centrosymmetric Tecto‐Borosulfate: Introduction of Various Molecular Cations into BiX[B(SO4)2]4 (X = H3O+, NO2 +, NH4 +, NO+)
Source: Chemistry. 2025 Oct 5;31(61):e02439. doi: 10.1002/chem.202502439 (PMC12587024; doi:10.1002/chem.202502439)
Supplement: Supplementary file 1 — Supporting Information [file CHEM-31-e02439-s001.pdf]

Variability of a Non-Centrosymmetric Tecto-Borosulfate: Introduction of Various Molecular Cations into $BiX[B(SO_4)_2]_4$ ($X = H_3O^+, NO_2^+, NH_4^+, NO^+$)

Supplements

Erich Turgunbajew, Matthias Hämmer, Lkhamsuren Bayarjargal, Florian Pielnhofer, Henning A. Höppe*

Table S1. Crystal data and structural refinement of $BiX[B(SO_4)_2]_4$ (X = NH_4^+, H_3O^+, NO_2^+, NO^+). Standard deviation is given in parentheses.

	**$Bi(NH_4)[B(SO_4)_2]_4$**	**$Bi(H_3O)[B(SO_4)_2]_4$**	**$Bi(NO_2)[B(SO_4)_2]_4$**	**$Bi(NO_2)_{0.236(4)}(NH_4)_{0.764(4)}[B(SO_4)_2]_4$**	**$Bi(NO)[B(SO_4)_2]_4$**
temperature/ K	250(2)	200(2)	200(2)	200(2)	200(2)
molar mass / g·mol^{-1}	1038.74	1039.72	1066.71	1066.71	1050.71
crystal system	tetragonal	tetragonal	tetragonal	tetragonal	tetragonal
crystal size / mm^3	0.09 x 0.05 x 0.04	0.07 x 0.08 x 0.12	0.11 x 0.11 x 0.06	0.11 x 0.11 x 0.06	0.03 x 0.06 x 0.07
space group	$I\bar{4}$ (Nr. 82)	$I\bar{4}$ (Nr. 82)	$I\bar{4}$ (Nr. 82)	$I\bar{4}$ (Nr. 82)	$I\bar{4}$ (Nr. 82)
a / Å	11.8746(1)	11.8428(10)	11.9081(1)	11.8934(3)	11.8277(6)
c / Å	8.1360(1)	8.1586(6)	8.1160(2)	8.1397(3)	8.1315(6)
volume / Å^3	1147.2(2)	1144.3(2)	1150.87(4)	1151.38(7)	1137.55(14)
Z	2	2	2	2	2
ρ_{calc} / g·cm^{-3}	3.007	3.021	3.078	3.013	3.068
absorption coefficient μ / mm^{-1}	8.568	8.592	8.551	8.539	8.646
F(000) / e	996	998	1020	1000	1004
Flack parameter	0.013(3)	BASF = 0.051(5)	0.019(3)	0.013(4)	BASF = 0.037(5)
radiation wavelength λ / Å	0.71073	0.71073	0.71073	0.71073	0.71073
absorption correction	multi-scan	multi-scan	multi-scan	multi-scan	multi-scan
transmission factor (min./max.)	0.6280 / 0.7192	0.5287 / 0.6908	0.5954 / 0.7493	0.6188 / 0.6992	0.5202 / 0.6642
Index range $h/k/l$	-21/21 | -21/21 | -14/14	-15/21 | -21/20 | -14/14	-19/19 | -19/19 | -13/13	-21/20 | -21/21 | -14/14	-19/16 |-19/19 | -13/13
theta range / °	2.426 – 39.982	2.432 – 39.999	2.419 – 34.968	2.422 – 39.978	2.435 – 34.996
reflections collected	28989	11012	18890	17500	12460
independent reflections	3487	3330	2382	3524	2384
observed reflections	3438	3315	2362	3410	2375
refined parameters	113	112	109	109	110
R_{int}	0.074	0.043	0.043	0.081	0.051
R_σ	0.049	0.058	0.046	0.095	0.054
R_1 (all data)	0.020	0.025	0.016	0.032	0.026
wR_2 (all data)	0.033	0.053	0.036	0.048	0.053
Goof	0.809	0.0910	0.87	0.80	0.892
residual electron density (min./max.) / e$^-$ Å^{-3}	-0.46 / 0.89	-0.99 / 2.74	-0.49 / 0.44	-0.49 / 0.44	-0.97 / 1.47
CSD no.	2477389	2477390	2410848	2477442	2477391

Table S2. Wyckoff symbol, atomic coordinates x; y; z and equivalent isotropic displacement parameters U_{eq} for $Bi(NO_2)[B(SO_4)_2]_4$ (corresponding standard deviations given in parentheses)

Atom	Wyckoff Symbol	Site	*x*	*y*	*z*	U_{eq} / Å^2
Bi	2a	-4	0	0	0	0.00540(4)
S1	8g	1	-0.19685(5)	0.02800(5)	0.32201(6)	0.00462(9)
O11	8g	1	-0.28966(18)	-0.0075(2)	0.2250(2)	0.0101(3)
O12	8g	1	-0.10220(16)	0.07821(17)	0.2382(2)	0.0080(3)
O13	8g	1	-0.24235(15)	0.10997(16)	0.4497(2)	0.0065(3)
O14	8g	1	-0.14729(16)	-0.06889(16)	0.4225(2)	0.0069(3)
S2	8g	1	-0.26083(5)	0.13300(5)	-0.16137(7)	0.00569(9)
O21	8g	1	-0.3180(2)	0.0349(2)	-0.2097(3)	0.0149(4)
O22	8g	1	-0.15159(18)	0.11829(19)	-0.0852(3)	0.0117(3)
O23	8g	1	-0.33488(16)	0.19959(16)	-0.0435(2)	0.0067(3)
O24	8g	1	-0.24527(16)	0.21695(16)	-0.3042(2)	0.0071(3)
B1	8g	1	-0.1804(2)	0.1943(2)	0.5460(3)	0.0060(4)
N1	2d	-4	-0.5	0	0.25	0.0168(9)
O3	4f	2	-0.5	0	0.1133(5)	0.0208(6)

Table S3. Anisotropic displacement parameters U_{ij} in Å^2 for $Bi(NO_2)[B(SO_4)_2]_4$ (corresponding standard deviations given in parentheses)

Atom	U_{11}	U_{22}	U_{33}	U_{12}	U_{13}	U_{23}
Bi	0.00558(4)	0.00558(4)	0.00503(5)	0.00000	0.00000	0.00000
S1	0.0051(2)	0.0051(2)	0.00362(17)	-0.00025(17)	0.00024(16)	-0.00031(16)
O11	0.0082(8)	0.0126(9)	0.0095(7)	-0.0011(7)	-0.0048(6)	-0.0028(7)
O12	0.0084(7)	0.0086(7)	0.0069(6)	-0.0010(6)	0.0049(6)	0.0008(6)
O13	0.0050(6)	0.0081(7)	0.0065(5)	0.0002(5)	0.0004(5)	-0.0038(5)
O14	0.0078(7)	0.0049(7)	0.0081(7)	-0.0015(5)	-0.0021(6)	0.0027(5)
S2	0.0069(2)	0.0054(2)	0.00482(18)	0.00191(17)	0.00063(17)	0.00017(16)
O21	0.0207(11)	0.0078(8)	0.0163(8)	-0.0031(8)	0.0029(8)	-0.0055(7)
O22	0.0087(8)	0.0166(9)	0.0098(7)	0.0055(7)	-0.0005(6)	0.0045(7)
O23	0.0070(7)	0.0069(7)	0.0062(5)	0.0008(6)	0.0031(5)	-0.0014(5)
O24	0.0096(7)	0.0078(7)	0.0038(6)	0.0034(6)	0.0019(6)	0.0008(6)
B1	0.0065(10)	0.0067(9)	0.0048(7)	0.0013(8)	-0.0004(7)	-0.0008(7)
N1	0.0101(12)	0.0101(12)	0.030(3)	0.00000	0.00000	0.00000
O3	0.0138(14)	0.0216(16)	0.0270(16)	0.0002(12)	0.00000	0.00000

Table S4. Wyckoff symbol, atomic coordinates x; y; z and equivalent isotropic displacement parameters U_{eq} for $Bi(NH_4)[B(SO_4)_2]_4$ (corresponding standard deviations given in parentheses)

**Atom**	**Wyckoff Symbol**	**Site**	**S.O.F**	***x***	***y***	***z***	U_{eq} / Å^2
Bi1	2*a*	-4		0.0	0.0	0.0	0.00721(3)
S1	8*g*	1		-0.02728(4)	-0.20005(4)	0.32093(6)	0.00850(8)
O11	8*g*	1		0.0091(2)	-0.29418(17)	0.2282(2)	0.0178(3)
O12	8*g*	1		-0.07654(15)	-0.10535(15)	0.23513(19)	0.0119(3)
O13	8*g*	1		-0.11114(15)	-0.24298(14)	0.44864(18)	0.0113(3)
O14	8*g*	1		0.06799(14)	-0.14915(14)	0.42382(19)	0.0102(2)
S2	8*g*	1		-0.36638(5)	-0.23886(5)	0.33404(6)	0.01152(9)
O21	8*g*	1		-0.46375(18)	-0.1807(2)	0.2832(3)	0.0229(4)
O22	8*g*	1		-0.3833(2)	-0.34828(18)	0.4100(2)	0.0226(4)
O23	8*g*	1		-0.30150(15)	-0.16458(14)	0.45312(19)	0.0115(3)
O24	8*g*	1		-0.28160(15)	-0.25473(15)	0.19310(18)	0.0114(3)
B1	8*g*	1		-0.1948(2)	-0.1801(2)	0.5426(3)	0.0100(4)
N1	4*f*	2	0.5	-0.5000	0.0	0.5192(13)	0.0223(16)
H1	8*g*	1	0.5	-0.5688(15)	-0.013(6)	0.445(3)	0.027
H2	8*g*	1	0.5	-0.512(6)	0.0691(14)	0.591(3)	0.027

Table S5. Anisotropic displacement parameters U_{ij} in Å^2 for $Bi(NH_4)[B(SO_4)_2]_4$ (corresponding standard deviations given in parentheses)

**Atom**	U_{11}	U_{22}	U_{33}	U_{12}	U_{13}	U_{23}
Bi1	0.00751(3)	0.00751(3)	0.00661(4)	0	0	0
S1	0.0108(2)	0.00891(19)	0.00575(16)	-0.00004(13)	0.00026(14)	0.00004(15)
O11	0.0250(10)	0.0142(8)	0.0142(7)	-0.0067(6)	0.0051(7)	-0.0003(7)
O12	0.0103(6)	0.0155(7)	0.0098(6)	0.0058(5)	-0.0009(5)	0.0003(5)
O13	0.0157(7)	0.0091(6)	0.0091(5)	0.0008(4)	0.0036(5)	-0.0022(5)
O14	0.0081(6)	0.0126(6)	0.0099(5)	-0.0023(5)	-0.0027(4)	0.0038(5)
S2	0.0127(2)	0.0150(2)	0.00682(17)	-0.00171(15)	0.00048(15)	-0.00702(17)
O21	0.0125(8)	0.0355(12)	0.0207(8)	-0.0086(8)	-0.0039(7)	-0.0003(8)
O22	0.0368(12)	0.0182(8)	0.0127(7)	-0.0015(6)	0.0083(7)	-0.0170(8)
O23	0.0137(7)	0.0126(7)	0.0082(5)	-0.0029(5)	-0.0023(5)	-0.0028(5)
O24	0.0136(7)	0.0147(7)	0.0061(5)	-0.0024(5)	0.0005(5)	-0.0059(5)
B1	0.0143(10)	0.0100(8)	0.0059(6)	0.0002(6)	0.0007(6)	-0.0049(7)
N1	0.018(2)	0.016(2)	0.033(5)	0	0	0.0045(18)
H1	-	-	-	-	-	-
H2	-	-	-	-	-	-

Table S6. Wyckoff symbol, atomic coordinates x; y; z and equivalent isotropic displacement parameters U_{eq} for $Bi(H_3O)[B(SO_4)_2]_4$ (corresponding standard deviations given in parentheses)

**Atom**	**Wyckoff Symbol**	**Site**	**S.O.F.**	***x***	***y***	***z***	**U_{eq} / Å^2**
Bi1	2*a*	-4		0.0	0.0	0.0	0.00641(4)
S1	8*g*	1		0.29955(7)	0.52626(7)	-0.1800(10)	0.00846(13)
O11	8*g*	1		0.2056(3)	0.4892(3)	-0.2733(4)	0.0184(6)
O12	8*g*	1		0.3937(2)	0.5775(2)	-0.2660(3)	0.0118(4)
O13	8*g*	1		0.3910(2)	0.2560(2)	0.0510(3)	0.0107(4)
O14	8*g*	1		0.3529(2)	0.4308(2)	-0.0793(3)	0.0096(4)
S2	8*g*	1		0.13360(7)	0.26261(7)	0.16260(9)	0.00770(12)
O21	8*g*	1		0.0349(3)	0.3228(3)	0.2080(4)	0.0182(6)
O22	8*g*	1		0.1162(3)	0.1523(2)	0.0903(4)	0.0154(5)
O23	8*g*	1		0.2004(2)	0.3347(2)	0.0437(3)	0.0091(4)
O24	8*g*	1		0.2158(2)	0.2485(2)	0.3067(3)	0.0105(4)
B1	8*g*	1		0.3073(3)	0.3188(3)	-0.0446(5)	0.0089(5)
O1	4*f*	2	0.5	0.0	0.5	-0.002(3)	0.0308(18)
H1	8*g*	1	0.5	0.072(4)	0.523(13)	-0.066(4)	0.037
H2	4*f*	2		0.0	0.5	0.124(4)	0.037

Table S7. Anisotropic displacement parameters U_{ij} in Å^2 for $Bi(H_3O)[B(SO_4)_2]_4$ (corresponding standard deviations given in parentheses)

**Atom**	**U_{11}**	**U_{22}**	**U_{33}**	**U_{12}**	**U_{13}**	**U_{23}**
Bi1	0.00710(5)	0.00710(5)	0.00501(6)	0	0	0
S1	0.0102(3)	0.0076(3)	0.0076(3)	-0.0006(2)	0.0005(2)	-0.0002(2)
O11	0.0168(13)	0.0153(13)	0.0232(15)	-0.0055(11)	-0.0077(11)	0.0006(10)
O12	0.0156(11)	0.0101(10)	0.0095(9)	0.0010(8)	0.0057(9)	-0.0020(8)
O13	0.0105(10)	0.0094(10)	0.0122(9)	-0.0031(8)	-0.0031(8)	0.0001(8)
O14	0.0115(10)	0.0070(9)	0.0102(9)	0.0010(7)	0.0002(8)	-0.0023(8)
S2	0.0081(3)	0.0094(3)	0.0057(3)	0.0009(2)	0.0002(2)	-0.0017(2)
O21	0.0104(12)	0.0250(15)	0.0193(13)	0.0009(11)	0.0038(10)	0.0032(11)
O22	0.0228(14)	0.0112(11)	0.0120(10)	-0.0007(9)	-0.0042(10)	-0.0071(10)
O23	0.0109(10)	0.0106(10)	0.0059(7)	0.0021(7)	0.0023(7)	-0.0006(8)
O24	0.0126(11)	0.0131(10)	0.0059(8)	0.0028(8)	-0.0023(8)	-0.0062(8)
B1	0.0110(14)	0.0097(14)	0.0061(10)	-0.0003(10)	-0.0002(10)	-0.0023(11)
O1	0.034(5)	0.020(4)	0.039(5)	0	0	0.004(3)
H1	-	-	-	-	-	-
H2	-	-	-	-	-	-

Table S8. Wyckoff symbol, atomic coordinates x; y; z and equivalent isotropic displacement parameters U_{eq} for $Bi(NO)[B(SO_4)_2]_4$ (corresponding standard deviations given in parentheses)

Atom	Wyckoff Symbol	Site	S.O.F.	*x*	*y*	*z*	U_{eq} / Å^2
Bi1	*2a*	-4		0.0000	0.0000	0.0000	0.00591(6)
S1	*2a*	1		-0.02730(8)	0.20014(8)	0.32132(10)	0.00621(15)
O11	8*g*	1		0.0090(3)	0.2947(3)	0.2287(4)	0.0140(6)
O12	8*g*	1		-0.0777(3)	0.1057(3)	0.2352(3)	0.0095(5)
O13	8*g*	1		0.2440(2)	0.1104(3)	0.5501(3)	0.0084(5)
O14	8*g*	1		0.0689(2)	0.1483(2)	0.4222(3)	0.0078(5)
S2	8*g*	1		0.26118(8)	0.13308(8)	0.16338(11)	0.00686(15)
O21	8*g*	1		0.3189(3)	0.0344(3)	0.2114(4)	0.0160(6)
O22	8*g*	1		0.1504(3)	0.1177(3)	0.0891(4)	0.0128(6)
O23	8*g*	1		0.3351(2)	0.1987(2)	0.0444(3)	0.0076(5)
O24	8*g*	1		0.2469(3)	0.2176(2)	0.3054(3)	0.0084(5)
B1	8*g*	1		0.1805(4)	0.1948(4)	0.4554(5)	0.0074(7)
O1	4f	2	0.5	0.5	0	-0.021(3)	0.050(5)
N1	4f	2	0.5	0.5	0	-0.144(4)	0.048(6)

Table S9. Anisotropic displacement parameters U_{ij} in Å^2 for $Bi(NO)[B(SO_4)_2]_4$ (corresponding standard deviations given in parentheses)

Atom	U_{11}	U_{22}	U_{33}	U_{12}	U_{13}	U_{23}
Bi1	0.00641(7)	0.00641(7)	0.00491(8)	0.000	0.000	0.000
S1	0.0060(4)	0.0070(4)	0.0057(3)	-0.0002(3)	0.0007(3)	-0.0001(3)
O11	0.0144(16)	0.0106(14)	0.0171(15)	0.0047(11)	0.0050(12)	0.0000(11)
O12	0.0080(12)	0.0128(13)	0.0078(11)	-0.0053(9)	-0.0007(9)	-0.0021(9)
O13	0.0070(12)	0.0095(12)	0.0088(10)	0.0033(9)	0.0022(8)	-0.0002(9)
O14	0.0062(12)	0.0089(12)	0.0081(11)	0.0009(9)	-0.0019(9)	-0.0005(9)
S2	0.0081(4)	0.0067(4)	0.0059(3)	0.0000(3)	0.0008(3)	-0.0017(3)
O21	0.0216(18)	0.0087(14)	0.0178(15)	0.0045(11)	0.0033(12)	0.0027(12)
O22	0.0102(13)	0.0187(15)	0.0096(11)	-0.0056(10)	-0.0003(10)	-0.0063(11)
O23	0.0082(12)	0.0075(12)	0.0072(10)	0.0022(8)	0.0028(8)	-0.0017(9)
O24	0.0114(13)	0.0087(12)	0.0051(10)	-0.0014(9)	0.0017(9)	-0.0032(9)
B1	0.0079(17)	0.0079(17)	0.0065(13)	0.0000(11)	0.0009(11)	-0.0014(12)
O1	0.029(7)	0.070(11)	0.050(13)	0	0	0.029(6)
N1	0.007(6)	0.029(8)	0.109(18)	0	0	0.001(5)

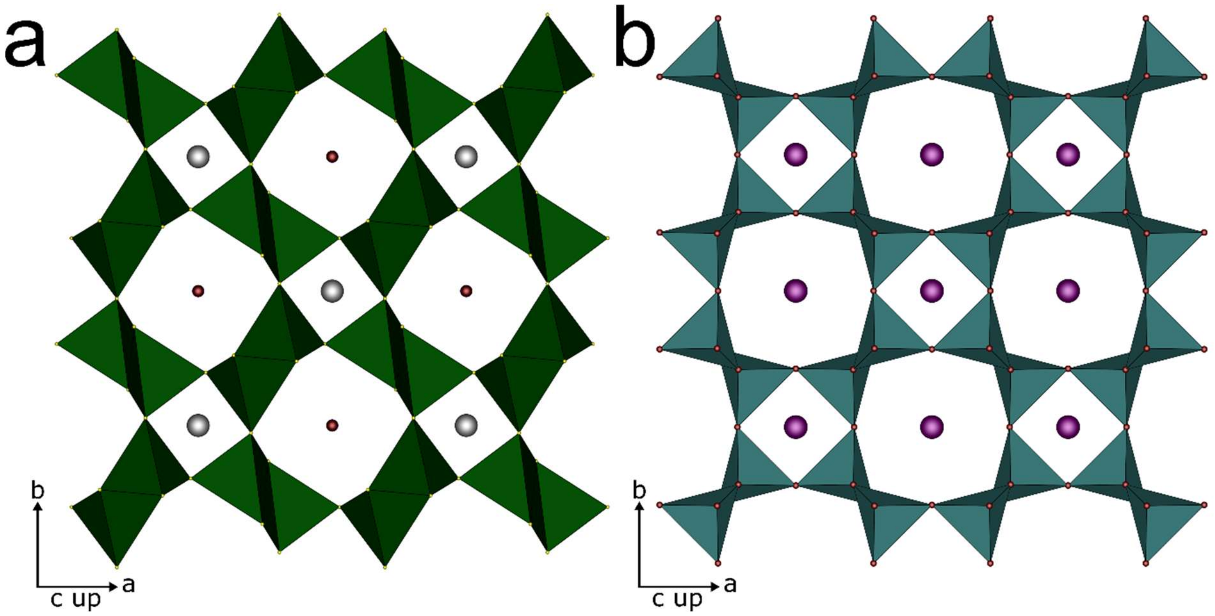

Figure S1. (a) Crystal structure of $Bi(NO_2)[B(SO_4)_2]_4$ depicted as connected BS_4 supertetrahedra (green) forming Vierer and Achter rings that lead to channels along the [001] direction; the NO^+ (red) and Bi^{3+} (grey) are located inside the channels. (b) Crystal structure of the structurally related tectosilicate $K_{1.14}Mg_{0.57}Si_{1.43}O_4$ analogously forming Vierer and Achter rings; Si/MgO_4 tetrahedra are coloured in petrol and potassium in purple.

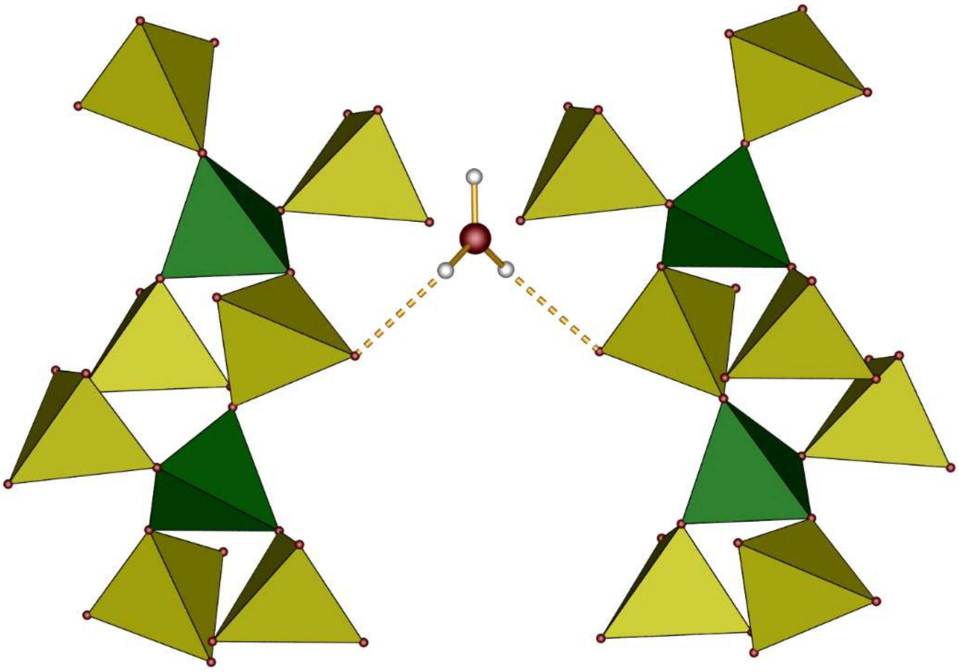

Figure S2. Hydrogen bonds of oxonium towards the borosulfate anion in in $Bi(H_3O)[B(SO_4)_2]$.

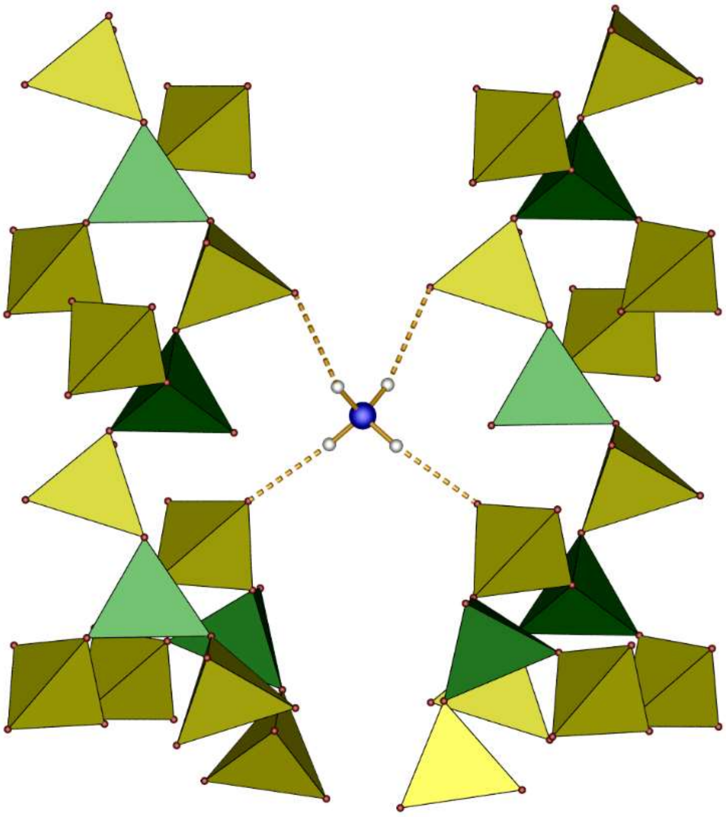

Figure S3. Hydrogen bonds of ammonium towards the borosulfate anion in $Bi(NH_4)[B(SO_4)_2]$.

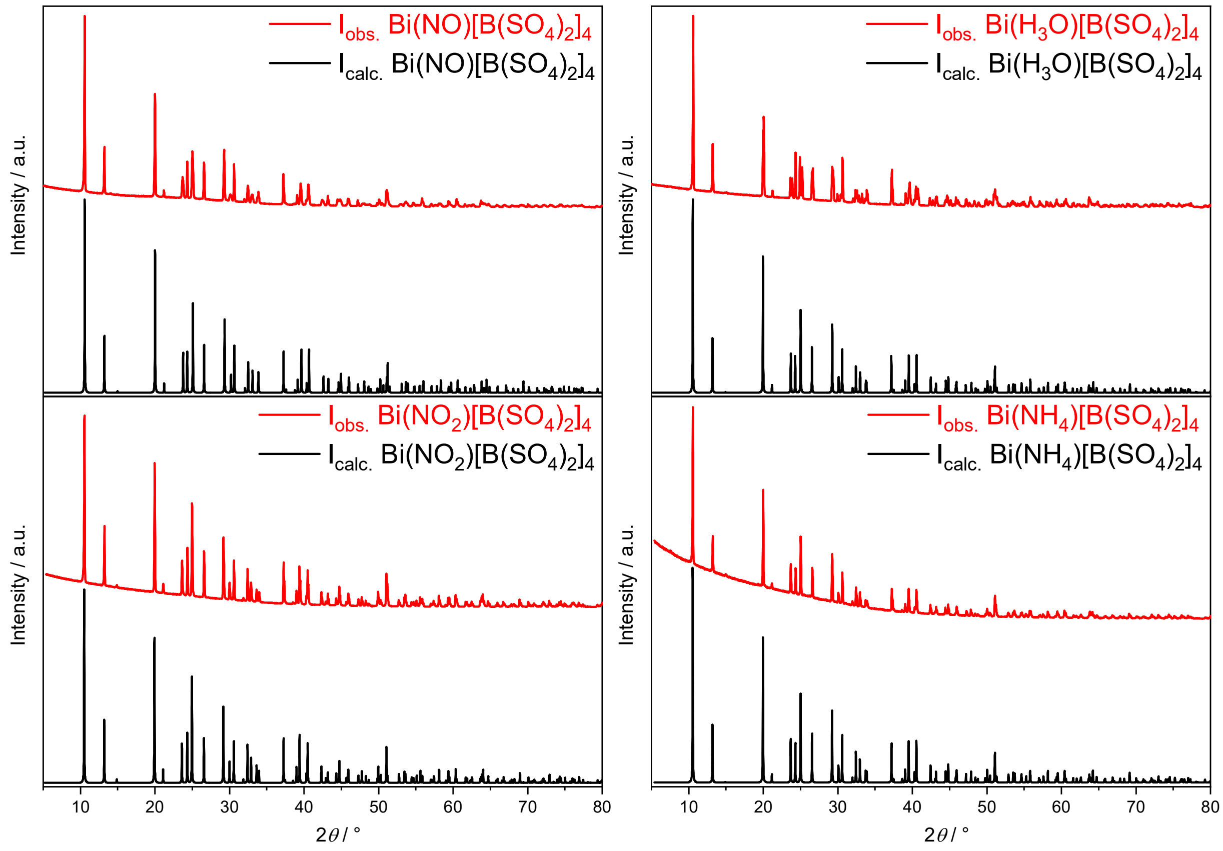

Figure S4. Calculated and observed PXRD patterns of $BiX[B(SO_4)_2]_4$ (X = NH_4^+, H_3O^+, NO_2^+, NO^+).

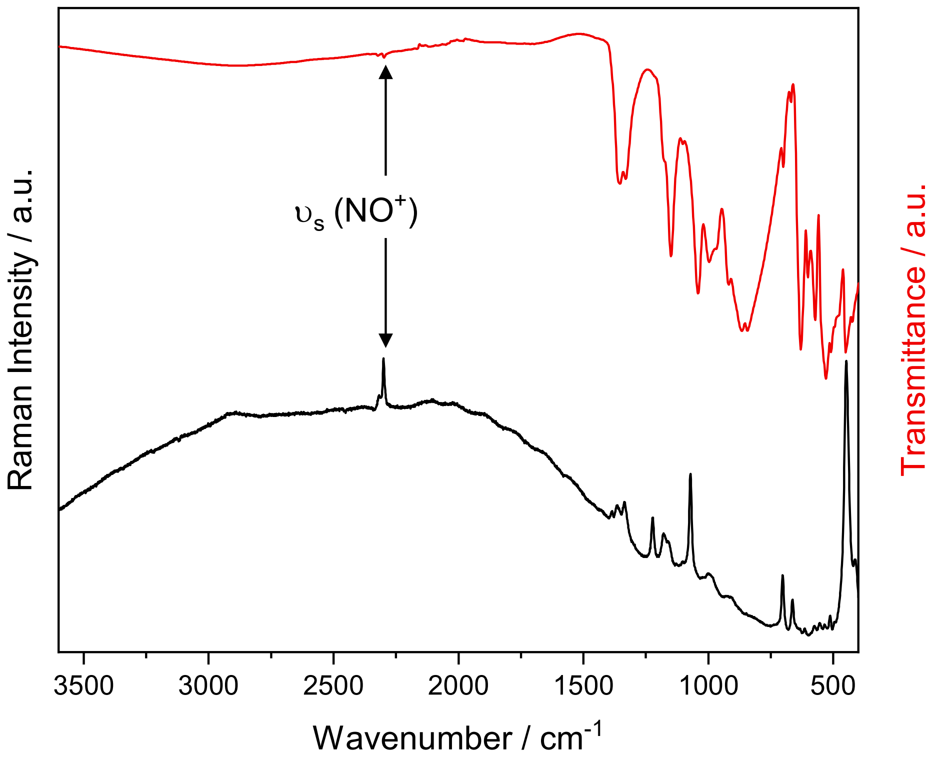

Figure S5. Experimental IR (top, red) and raman spectrum (bottom, black) of $Bi(NO)[B(SO_4)_2]_4$.

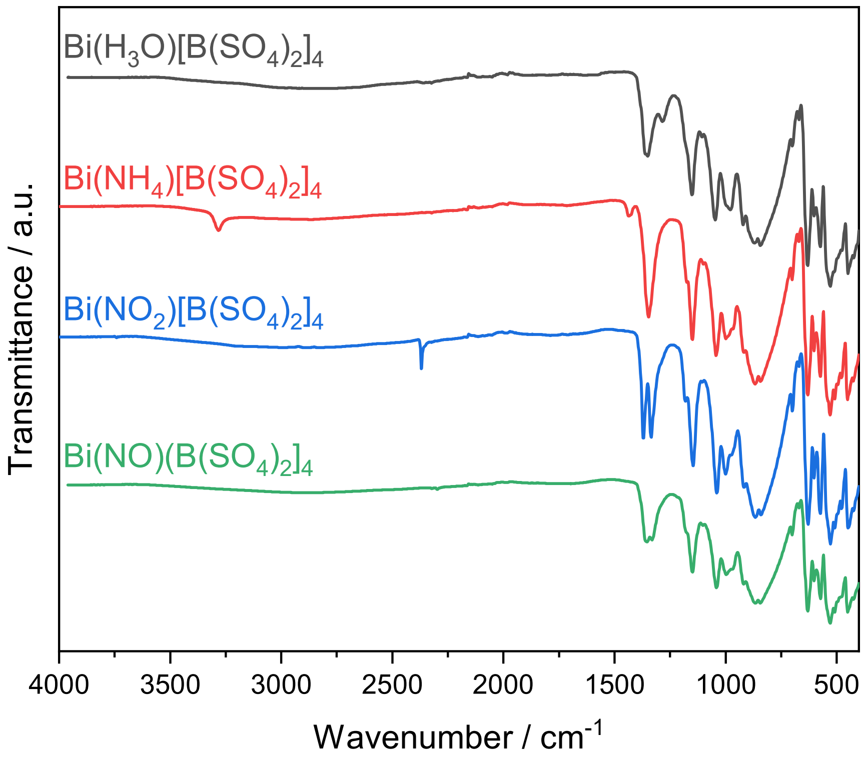

Figure S6. Infrared spectra of $BiX[B(SO_4)_2]_4$ (X = NH_4^+, H_3O^+, NO_2^+, NO^+).

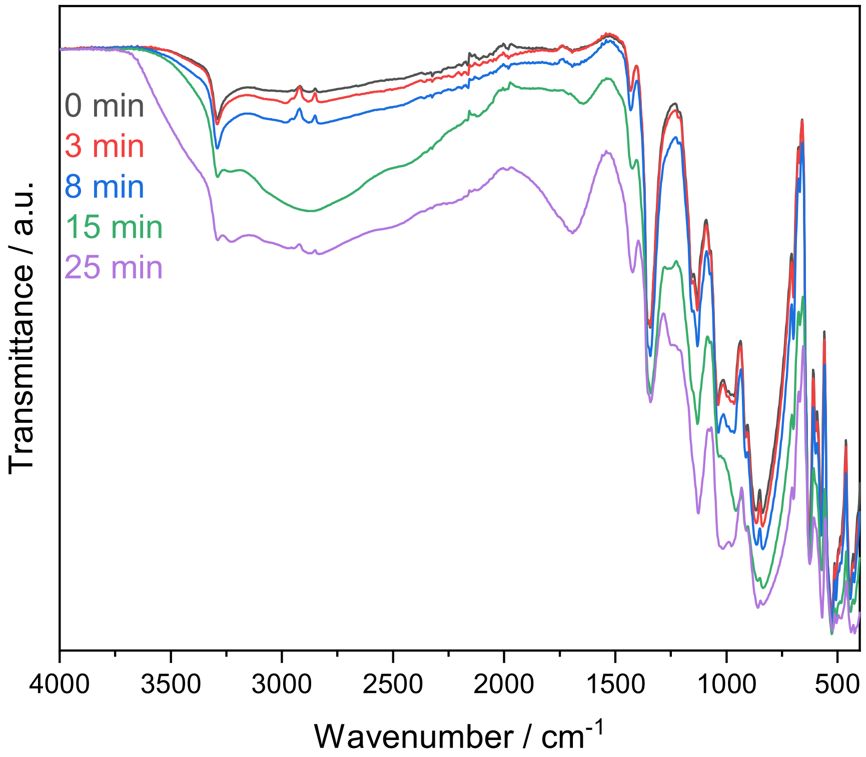

Figure S7. Infrared spectra of $Bi(NH_4)[B(SO_4)_2]_4$ exposed to air for different periods of time.

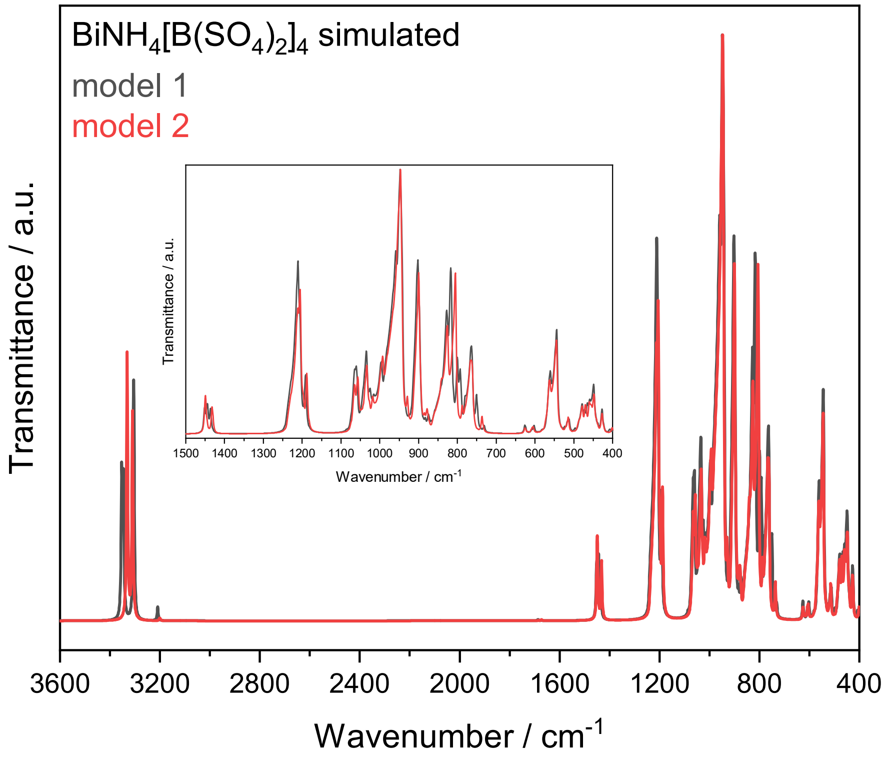

Figure S8. Simulated infrared spectra of two fully ordered models of $BiNH_4[B(SO_4)_2]_4$ (model 1 in grey; model 2 in red) in the range between 3600 – 400 cm^{-1}, with an inset in the range between 1500 – 400 cm^{-1}.

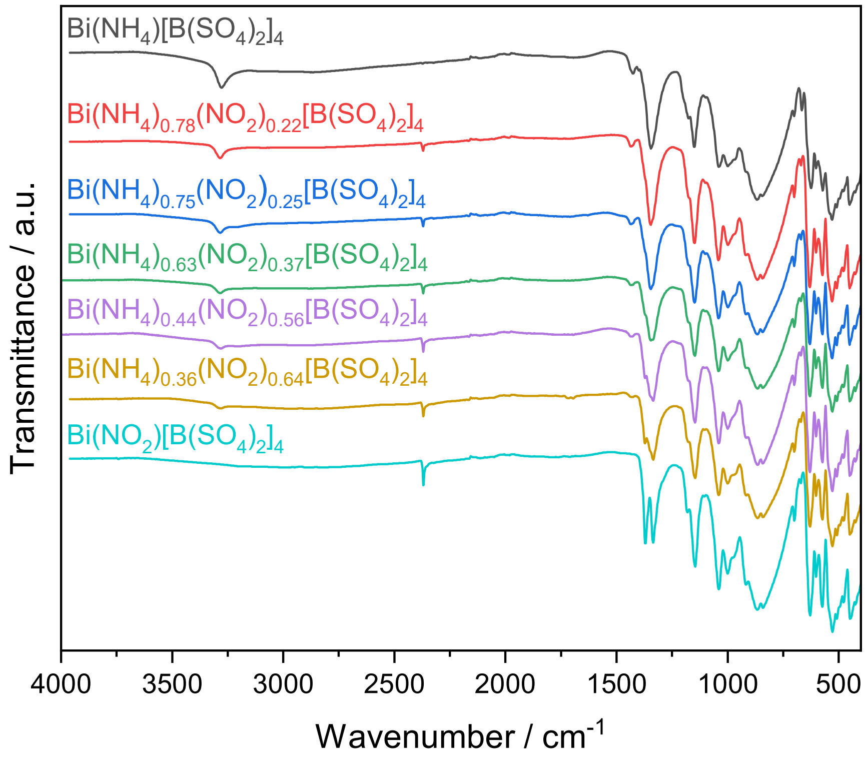

Figure S9. Infrared spectra of $Bi(NH_4)_x(NO_2)_{1-x}[B(SO_4)_2]_4$ with varying ratio of ammonium to nitronium cations. The composition was determined by SC-XRD measurements averaging the results of 3 single crystals.

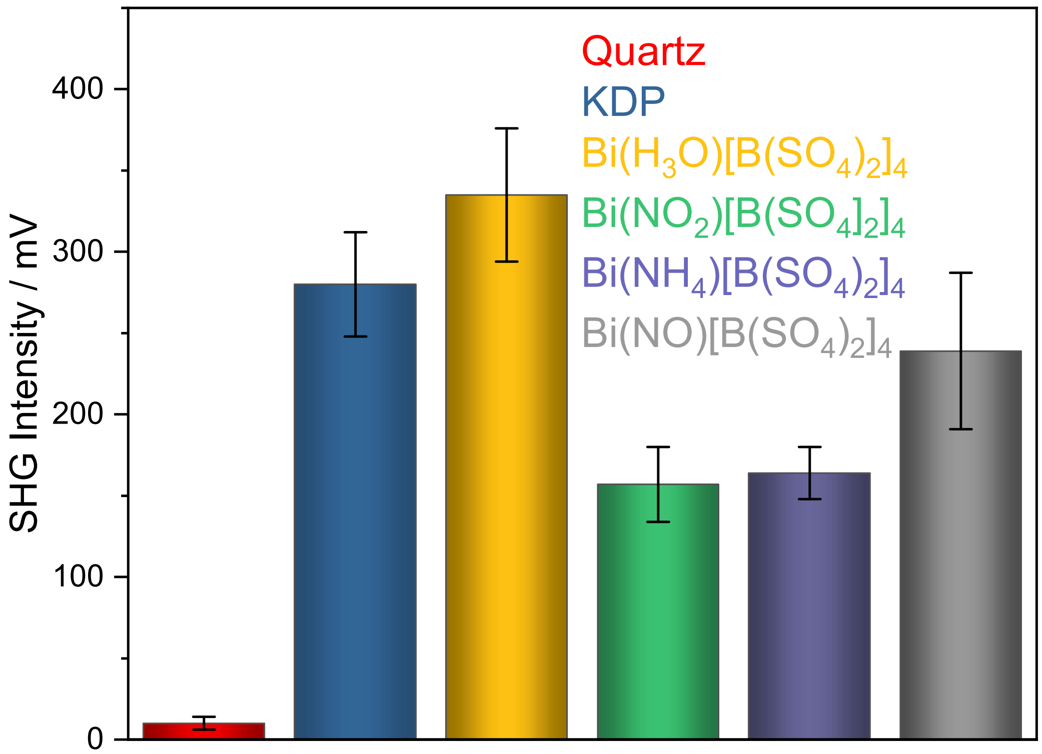

Figure S10. SHG intensities of Bi$X[B(SO_4)_2]_4$ (X = NH_4^+, H_3O^+, NO_2^+, NO^+), the reference material KDP and quartz recorded for the grain size range 25-50 µm.

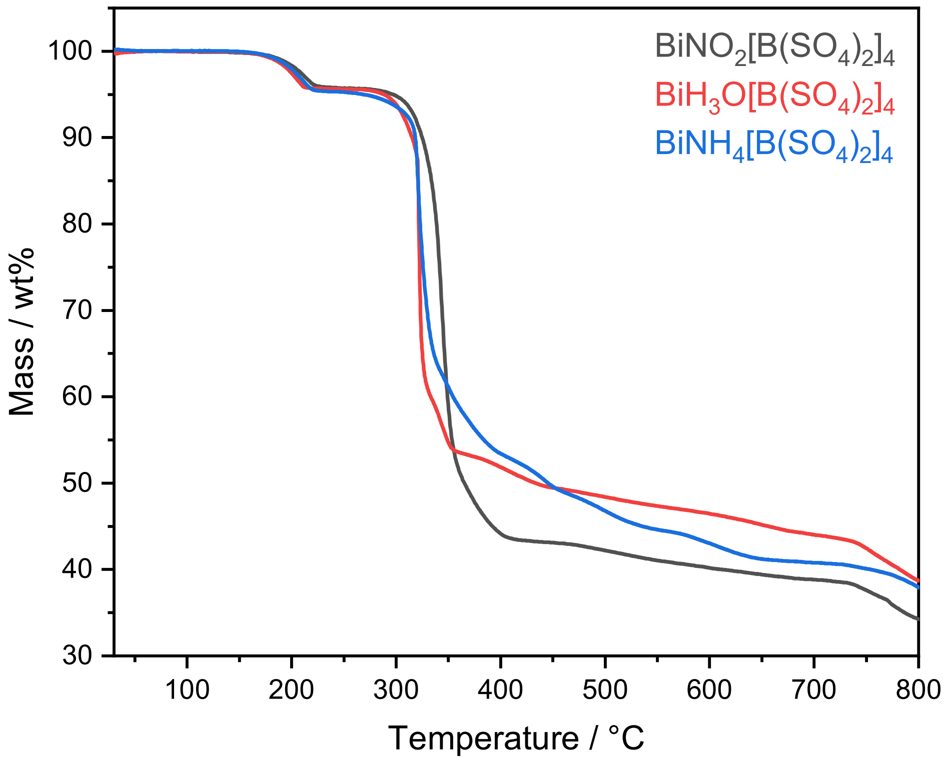

Figure S11. Thermogravimetric analyses of $BiX[B(SO_4)_2]_4$ (X = NO_2 (grey), H_3O (red), NH_4 (blue)).

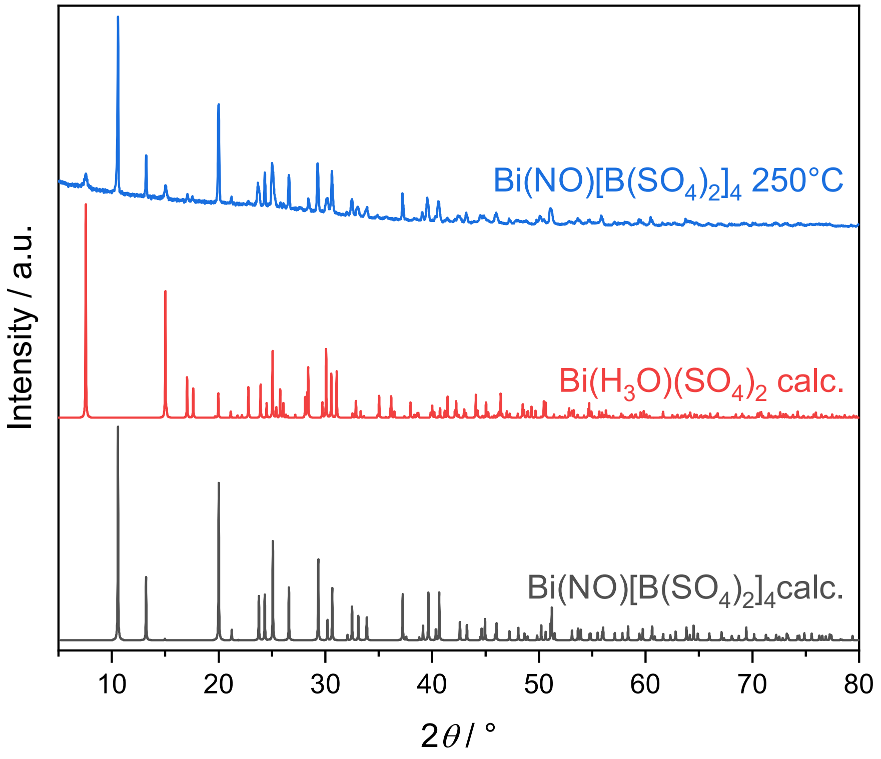

Figure S12. Experimental PXRD pattern of $Bi(NO)[B(SO_4)_2]_4$ (blue) after one hour at 250°C in a nitrogen atmosphere compared to calculated patters of $Bi(NO)[B(SO_4)_2]_4$ (grey) and $Bi(H_3O)(SO_4)_2$ (red).

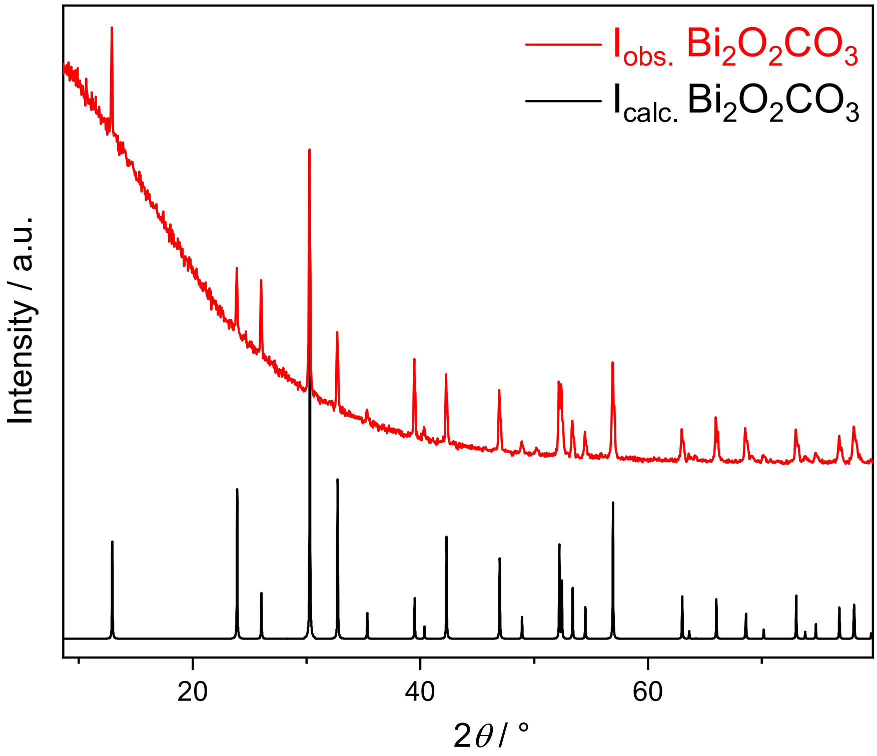

Figure S13. Calculated (black) and observed (red) PXRD patterns of the precursor $Bi_2(CO_3)O_2$.

Table S10. Electrostatic calculations based on the *MAPLE* concept. [1]

used compounds	MAPLE / kJ·mol^{-1}
NH_3	10054
N_2O_5	48133
Bi_2O_3	14379
B_2O_3	21924
SO_3	29930
NH_3 + 0.5 Bi_2O_3 + 2 B_2O_3 + 8 SO_3 + 0.5 H_2O	301899
$Bi(NH_4)[B(SO_4)_2]_4$	298780
Δ = 1.02 %	
0.5 N_2O_5 + 0.5 Bi_2O_3 + 2 B_2O_3 + 7,5 SO_3	314547
$Bi(NO_2)[B(SO_4)_2]_4$	312233
Δ = 0.74 %	
1.5 H_2O + 0.5 $Bi_2(SO_4)_3$ + 2 B_2O_3 + 6.5 SO_3	298620
$Bi(H_3O)[B(SO_4)_2]_4$	295619
Δ = 1.00 %	

Table S11. Geometric parameters and calculated eccentricity for $BiX[B(SO_4)_2]_4$ (X = NH_4^+, NO_2^+, NO^+, H_3O^+) and the reference $BiVO_4$.

**Bi*X*[B(SO4)$_2$]$_4$, *X* =**	**NO_2**	**NH_4**	**H_3O**	**NO**	**$BiVO_4$**
distance of central atom to centroid R_c / Å	0.00037	0.0005	0	0.00031	0.29
CN	8	8	8	8	8
Diameter of surrounding sphere / Å	2.467	2.459	2.463	2.464	2.536
eccentricity	0.0001	0.0002	0	0.0001	0.1143

Table S12. Deviations from tetrahedral symmetry in percent, calculated with the method of Balić-Žunić and Mackovicky.[2]

	$Bi(NO_2)[B(SO_4)_2]_4$	$Bi(NH_4)[B(SO_4)_2]_4$	$Bi(NO)[B(SO_4)_2]_4$
SO_4 (1)	-0.26	-0.27	-0.29
SO_4 (2)	-0.15	-0.13	-0.15
BO_4	-0.35	-0.31	-0.34

Table S13. Selected interatomic distances in $BiX[B(SO_4)_2]_4$ (*X* = NH_4^+, NO_2^+, NO^+). Standard deviations given in parentheses.

	$Bi(NO_2)[B(SO_4)_2]_4$	$Bi(NH_4)[B(SO_4)_2]_4$	$Bi(NO)[B(SO_4)_2]_4$
Bi – O_{term}	239.2(2) – 246.7(2)	238.8(2) – 245.9(2)	237.4(3) – 245.4(3)
Σ r_{ion} Bi – O	252		
S – O_{br}	152.3(2) – 154.1(2)	152.0(2) – 1.538(2)	151.8(3) – 153.7(3)
S – O_{term} (Bi^{3+})	144.5(2) – 145.1(2)	144.7(2) – 145.3(2)	144.6(3) – 145.3(3)
S – O_{term} (X^+)	140.8(2) – 142.1(2)	140.9(2) – 141.6(2)	140.7(3) – 141.6(3)
Σ r_{ion} S – O	147		
B – O	146.4(3) – 147.0(3)	145.9(3) – 147.5(3)	145.7(5) – 147.4(4)
Σ r_{ion} B – O	146		
O-S-O	101.8(10) – 117.9(12)	102.4(9) – 118.7(11)	102.2(17) – 118.6(18)
O-B-O	106.7(2) – 113.8(2)	107.2(19) – 113.1(19)	106.7(3) – 113.5(3)

Table S14. Thermal decomposition products of $BiX[B(SO_4)_2]_4$ (X = H_3O, NH_4, NO_2) after the first decomposition step in a (a) TGA and a (b) TPXRPD measurement, respectivly. All subsequent decompositon steps are discussed in the manuscript.

(a) TGA

$Bi(H_3O)[B(SO_4)_2]_4 \rightarrow 0.5\ Bi_2(SO_4)_3 + 2\ B_2O_3 + 1.5\ H_2O\ (g) + 6.5\ SO_3\ (g)$
$Bi(NH_4)[B(SO_4)_2]_4 \rightarrow 0.5\ Bi_2(SO_4)_3 + 2\ B_2O_3 + NH_3\ (g) + 6.5\ SO_3\ (g) + 0.5\ H_2O\ (g)$
$Bi(NO_2)[B(SO_4)_2]_4 \rightarrow 0.5\ Bi_2(SO_4)_3 + 2\ B_2O_3 + NO_2\ (g) + 6.5\ SO_3\ (g) + 0.25\ O_2\ (g)$

(b) TPPXRD

$Bi(H_3O)[B(SO_4)_2]_4 \rightarrow 0.5\ Bi_2[B_2(SO_4)_6] + 1.5\ B_2O_3 + 1.5\ H_2O\ (g) + 5\ SO_3\ (g)$
$Bi(NH_4)[B(SO_4)_2]_4 \rightarrow 0.5\ Bi_2[B_2(SO_4)_6] + 1.5\ B_2O_3 + NH_3\ (g) + 5\ SO_3\ (g) + 0.5\ H_2O\ (g)$
$Bi(NO_2)[B(SO_4)_2]_4 \rightarrow 0.5\ Bi_2[B_2(SO_4)_6] + 1.5\ B_2O_3 + NO\ (g) + 5\ SO_3\ (g) + 0.75\ O_2\ (g)$

Table S15. Calculated IR and Raman activ vibrations of $Bi(NO)[B(SO_4)_2]_4$.

Wavenumber / cm^{-1}	Irrep.	IR	Intensity / $km \cdot mol^{-1}$	Raman
-35	B	A	-636	A
0	A	A	0	A
0	B	A	0	A
0	B	A	0	A
67	B	A	-58	A
68	A	A	-73	A
70	B	A	-38	A
72	A	A	-17	A
76	A	A	-9	A
79	B	A	-52	A
80	A	A	0	A
84	B	A	-47	A
86	B	A	-51	A
87	A	A	-26	A
92	A	A	-108	A
99	A	A	0	A
100	B	A	-151	A
107	B	A	-247	A
110	A	A	-25	A
112	A	A	-69	A
120	B	A	-31	A
126	B	A	-8	A
130	A	A	-2	A
135	B	A	-24	A
138	B	A	-6	A
140	A	A	0	A
145	A	A	-3	A

157	A	A	-3	A
159	B	A	-9	A
161	B	A	-2	A
165	A	A	-61	A
177	B	A	-26	A
181	B	A	-9	A
198	A	A	0	A
206	B	A	-42	A
211	B	A	-1	A
212	A	A	-5	A
218	B	A	-95	A
222	B	A	-45	A
239	A	A	-121	A
243	A	A	0	A
248	A	A	-4	A
250	B	A	-9	A
251	A	A	-1	A
252	B	A	-1	A
262	A	A	-1	A
262	B	A	-33	A
264	B	A	-66	A
266	A	A	-1	A
281	B	A	-31	A
282	A	A	-67	A
284	B	A	-5	A
296	A	A	0	A
322	B	A	-46	A
324	B	A	-44	A
335	A	A	-109	A
356	A	A	-24	A
358	B	A	-32	A
361	B	A	-38	A
364	A	A	-58	A
367	A	A	-61	A
382	B	A	-113	A
384	B	A	-100	A
385	A	A	-8	A
401	B	A	-33	A
403	A	A	-1	A
406	B	A	-46	A
421	A	A	-13	A
423	A	A	-417	A
435	B	A	-74	A
436	A	A	-2	A
439	B	A	-106	A
441	A	A	-5	A
446	A	A	-1185	A

458	B	A	-195	A
461	B	A	-204	A
463	A	A	-6	A
465	B	A	-103	A
468	B	A	-151	A
472	A	A	-419	A
496	A	A	-6	A
511	B	A	-149	A
512	B	A	-122	A
513	A	A	-2	A
520	A	A	-31	A
536	A	A	-11	A
541	B	A	-1147	A
542	B	A	-1167	A
559	A	A	-650	A
562	A	A	-106	A
578	B	A	-13	A
580	B	A	-2	A
604	B	A	-45	A
606	A	A	-1	A
606	B	A	-42	A
624	A	A	-70	A
732	B	A	-52	A
736	B	A	-24	A
749	A	A	-500	A
756	A	A	-2344	A
763	A	A	-431	A
784	B	A	-107	A
788	B	A	-3	A
791	A	A	-38	A
805	B	A	-2106	A
808	B	A	-1948	A
825	A	A	-2	A
826	A	A	-3454	A
874	A	A	-97	A
885	A	A	-343	A
888	B	A	-706	A
888	B	A	-1632	A
890	B	A	-1404	A
892	B	A	-1921	A
893	A	A	0	A
896	A	A	-69	A
923	A	A	-3	A
935	A	A	-3529	A
938	B	A	-2053	A
940	B	A	-2218	A
955	A	A	-73	A

998	A	A	-322	A
1013	B	A	-3	A
1017	B	A	-14	A
1031	B	A	-244	A
1034	A	A	-73	A
1037	B	A	-173	A
1045	A	A	-13	A
1049	A	A	-4	A
1061	B	A	-241	A
1067	B	A	-41	A
1077	A	A	-11	A
1125	B	A	-2391	A
1128	A	A	-37	A
1138	B	A	-2087	A
1184	A	A	-28	A
1216	B	A	-876	A
1225	A	A	-135	A
1227	B	A	-767	A
1241	A	A	-32	A
2098	A	A	-177	A

Table S16. Calculated IR and Raman activ vibrations of $Bi(NO_2)[B(SO_4)_2]_4$.

Wavenumber / cm^{-1}	Irrep.	IR	Intensity / $km \cdot mol^{-1}$	Raman
0	B	A	0	A
0	E	A	0	A
63	E	A	114	A
64	A	I	0	A
64	B	A	1	A
70	B	A	202	A
75	A	I	0	A
80	E	A	25	A
87	B	A	61	A
94	E	A	70	A
97	B	A	168	A
98	A	I	0	A
113	A	I	0	A
114	E	A	312	A
118	B	A	35	A
124	E	A	218	A
133	B	A	0	A
137	B	A	1	A
141	E	A	66	A
143	A	I	0	A
156	A	I	0	A
157	E	A	22	A

159	B	A	68	A
168	E	A	31	A
176	E	A	31	A
198	A	I	0	A
206	B	A	0	A
207	E	A	7	A
240	B	A	130	A
243	A	I	0	A
248	E	A	8	A
249	B	A	0	A
250	A	I	0	A
258	A	I	0	A
259	E	A	55	A
265	A	I	0	A
278	E	A	54	A
279	B	A	122	A
296	A	I	0	A
324	E	A	86	A
333	B	A	147	A
356	B	A	23	A
359	E	A	194	A
364	B	A	137	A
366	A	I	0	A
381	E	A	5	A
381	A	I	0	A
389	E	A	641	A
402	E	A	39	A
404	A	I	0	A
418	B	A	22	A
426	B	A	554	A
436	A	I	0	A
439	E	A	72	A
439	A	I	0	A
444	B	A	1105	A
458	E	A	349	A
464	A	I	0	A
467	E	A	420	A
477	B	A	326	A
495	A	I	0	A
513	A	I	0	A
514	E	A	231	A
519	B	A	66	A
538	B	A	14	A
542	E	A	2268	A
561	B	A	694	A
566	A	I	0	A
580	E	A	4	A

603	E	A	121	A
604	A	I	0	A
622	B	A	76	A
730	E	A	69	A
747	A	I	0	A
750	B	A	2961	A
768	B	A	527	A
789	E	A	45	A
791	A	I	0	A
809	E	A	4847	A
823	B	A	2836	A
826	A	I	0	A
877	A	I	0	A
879	B	A	466	A
887	E	A	2586	A
892	A	I	0	A
894	B	A	337	A
895	E	A	3057	A
920	A	I	0	A
940	E	A	4380	A
946	B	A	3826	A
952	A	I	0	A
997	B	A	270	A
1014	E	A	1	A
1033	E	A	375	A
1033	B	A	91	A
1044	A	I	0	A
1046	B	A	5	A
1061	E	A	551	A
1078	A	I	0	A
1164	B	A	2	A
1175	E	A	2052	A
1207	A	I	0	A
1221	E	A	1646	A
1225	B	A	173	A
1240	A	I	0	A
1318	A	I	0	A
2310	B	A	242	A

Table S17. Calculated IR and Raman activ vibrations of $Bi(H_3O)[B(SO_4)_2]_4$ model 1.

Wavenumber / cm^{-1}	Irrep.	IR	Intensity / $km{\cdot}mol^{-1}$	Raman
-218	E	A	-1922	A
0	B	A	0	A
0	E	A	0	A
36	A	I	0	A

49	E	A	-8	A
59	E	A	-12	A
63	B	A	-22	A
66	E	A	-103	A
67	B	A	-47	A
69	A	I	0	A
75	E	A	-42	A
77	A	I	0	A
80	E	A	-34	A
83	B	A	-6	A
84	E	A	-91	A
85	B	A	-221	A
88	B	A	-1	A
90	A	I	0	A
92	A	I	0	A
92	B	A	-181	A
95	E	A	-111	A
96	B	A	-44	A
99	A	I	0	A
103	B	A	-37	A
104	E	A	-201	A
109	B	A	-16	A
110	A	I	0	A
110	E	A	-53	A
115	B	A	-3	A
118	E	A	-154	A
121	B	A	-45	A
122	A	I	0	A
124	E	A	-74	A
128	A	I	0	A
131	B	A	-67	A
134	E	A	-96	A
142	A	I	0	A
145	B	A	-3	A
150	A	I	0	A
152	E	A	-9	A
153	A	I	0	A
156	E	A	-9	A
156	B	A	-147	A
165	A	I	0	A
171	E	A	-11	A
183	E	A	-15	A
184	B	A	-2	A
191	B	A	-1	A
196	A	I	0	A
199	E	A	-97	A
200	A	I	0	A

208	B	A	-2	A
211	E	A	-10	A
215	B	A	-1	A
235	B	A	-147	A
235	A	I	0	A
237	E	A	-186	A
241	B	A	-63	A
242	E	A	-70	A
244	A	I	0	A
248	A	I	0	A
249	B	A	-45	A
249	E	A	-165	A
256	B	A	-10	A
257	E	A	-54	A
262	A	I	0	A
263	E	A	-211	A
266	A	I	0	A
267	A	I	0	A
274	E	A	-210	A
283	E	A	-160	A
283	B	A	-91	A
285	A	I	0	A
291	A	I	0	A
294	B	A	-23	A
297	A	I	0	A
311	E	A	-42	A
318	B	A	-13	A
326	E	A	-275	A
340	B	A	-219	A
342	A	I	0	A
353	B	A	-80	A
355	E	A	-104	A
357	E	A	-30	A
359	A	I	0	A
361	B	A	-114	A
362	A	I	0	A
365	B	A	-79	A
368	A	I	0	A
371	E	A	-65	A
378	A	I	0	A
379	B	A	-3	A
381	E	A	-269	A
402	A	I	0	A
404	E	A	-82	A
409	A	I	0	A
413	E	A	-5	A
415	B	A	-160	A

421	B	A	-41	A
432	E	A	-22	A
432	A	I	0	A
436	B	A	-1976	A
437	A	I	0	A
440	B	A	-948	A
440	E	A	-65	A
444	A	I	0	A
449	A	I	0	A
451	B	A	-538	A
453	E	A	-180	A
456	A	I	0	A
460	E	A	-730	A
465	E	A	-456	A
466	A	I	0	A
466	B	A	-2	A
468	E	A	-316	A
478	E	A	-15	A
478	B	A	-812	A
486	B	A	-35	A
495	A	I	0	A
499	E	A	-29	A
509	A	I	0	A
511	A	I	0	A
513	B	A	0	A
514	E	A	-558	A
518	B	A	-273	A
530	E	A	-137	A
535	B	A	-22	A
544	B	A	-18	A
544	E	A	-4416	A
553	A	I	0	A
557	B	A	-1041	A
560	A	I	0	A
567	E	A	-37	A
571	B	A	-29	A
581	E	A	-77	A
583	A	I	0	A
599	E	A	-165	A
600	A	I	0	A
607	E	A	-64	A
609	A	I	0	A
611	B	A	-4	A
621	B	A	-115	A
723	A	I	0	A
729	B	A	-249	A
738	E	A	-554	A

740	B	A	-872	A
742	A	I	0	A
752	E	A	-2081	A
758	B	A	-218	A
762	B	A	-232	A
763	E	A	-443	A
764	A	I	0	A
773	B	A	-5645	A
773	E	A	-794	A
780	A	I	0	A
794	E	A	-1168	A
800	A	I	0	A
803	B	A	-1258	A
806	E	A	-5507	A
818	A	I	0	A
822	B	A	-4324	A
824	A	I	0	A
838	E	A	-631	A
850	A	I	0	A
870	E	A	-1138	A
872	B	A	-809	A
889	E	A	-1144	A
889	A	I	0	A
890	B	A	-100	A
902	E	A	-295	A
906	A	I	0	A
907	B	A	-470	A
909	E	A	-6300	A
921	A	I	0	A
932	B	A	-471	A
934	A	I	0	A
934	E	A	-222	A
944	B	A	-6422	A
947	E	A	-9631	A
950	B	A	-546	A
959	A	I	0	A
984	E	A	-344	A
995	B	A	-835	A
997	A	I	0	A
1011	A	I	0	A
1027	E	A	-538	A
1036	B	A	-1	A
1036	E	A	-875	A
1046	B	A	-32	A
1047	A	I	0	A
1050	E	A	-247	A
1053	A	I	0	A

1056	B	A	-3	A
1066	B	A	-19	A
1066	E	A	-603	A
1073	B	A	-142	A
1078	E	A	-177	A
1079	A	I	0	A
1089	A	I	0	A
1125	E	A	-3071	A
1149	B	A	-41	A
1158	A	I	0	A
1192	B	A	-40	A
1192	E	A	-2245	A
1203	E	A	-826	A
1209	B	A	-198	A
1218	A	I	0	A
1222	A	I	0	A
1225	E	A	-1726	A
1240	A	I	0	A
1250	B	A	-73	A
1523	E	A	-151	A
1634	B	A	-283	A
1635	A	I	0	A
2797	E	A	-13154	A
2869	A	I	0	A
2871	B	A	-1614	A
3676	A	I	0	A
3676	B	A	-813	A

Table S18. Calculated IR and Raman active vibrations of $Bi(H_3O)[B(SO_4)_2]_4$ model 2.

Wavenumber / cm^{-1}	Irrep.	IR	Intensity / km·mol^{-1}	Raman
-219	E	A	-1924	A
0	B	A	0	A
0	E	A	0	A
37	A	I	0	A
49	E	A	-12	A
59	E	A	-10	A
64	B	A	-11	A
67	B	A	-65	A
67	E	A	-95	A
70	A	I	0	A
75	E	A	-43	A
76	A	I	0	A
80	E	A	-32	A
84	B	A	-1	A
84	E	A	-90	A

85	B	A	-221	A
88	B	A	-9	A
90	A	I	0	A
91	A	I	0	A
92	B	A	-171	A
95	E	A	-111	A
96	B	A	-43	A
99	A	I	0	A
103	B	A	-42	A
105	E	A	-191	A
109	B	A	-14	A
109	A	I	0	A
110	E	A	-51	A
115	B	A	-3	A
119	E	A	-157	A
121	B	A	-41	A
122	A	I	0	A
123	E	A	-73	A
129	A	I	0	A
131	B	A	-72	A
134	E	A	-103	A
142	A	I	0	A
145	B	A	-2	A
150	A	I	0	A
152	E	A	-9	A
153	A	I	0	A
156	E	A	-8	A
156	B	A	-146	A
165	A	I	0	A
171	E	A	-9	A
183	E	A	-14	A
184	B	A	-1	A
191	B	A	-1	A
196	A	I	0	A
199	E	A	-96	A
200	A	I	0	A
208	B	A	-2	A
211	E	A	-9	A
215	B	A	-1	A
235	B	A	-148	A
235	A	I	0	A
237	E	A	-191	A
241	B	A	-62	A
242	E	A	-72	A
244	A	I	0	A
248	A	I	0	A
249	B	A	-45	A

249	E	A	-162	A
256	B	A	-10	A
257	E	A	-56	A
262	A	I	0	A
263	E	A	-207	A
266	A	I	0	A
267	A	I	0	A
274	E	A	-208	A
283	E	A	-162	A
283	B	A	-90	A
285	A	I	0	A
291	A	I	0	A
294	B	A	-24	A
297	A	I	0	A
311	E	A	-44	A
318	B	A	-13	A
326	E	A	-284	A
340	B	A	-219	A
342	A	I	0	A
353	B	A	-71	A
355	E	A	-108	A
357	E	A	-28	A
359	A	I	0	A
361	B	A	-124	A
362	A	I	0	A
365	B	A	-80	A
368	A	I	0	A
371	E	A	-65	A
378	A	I	0	A
379	B	A	-3	A
381	E	A	-269	A
402	A	I	0	A
404	E	A	-80	A
409	A	I	0	A
413	E	A	-4	A
415	B	A	-162	A
421	B	A	-41	A
432	E	A	-23	A
432	A	I	0	A
435	B	A	-1926	A
437	A	I	0	A
440	B	A	-951	A
440	E	A	-67	A
445	A	I	0	A
449	A	I	0	A
451	B	A	-577	A
452	E	A	-183	A

456	A	I	0	A
460	E	A	-719	A
465	E	A	-440	A
465	A	I	0	A
466	B	A	-3	A
468	E	A	-342	A
478	E	A	-16	A
478	B	A	-816	A
486	B	A	-34	A
495	A	I	0	A
498	E	A	-21	A
509	A	I	0	A
510	A	I	0	A
513	B	A	0	A
513	E	A	-590	A
517	B	A	-275	A
530	E	A	-111	A
534	B	A	-20	A
544	E	A	-4429	A
544	B	A	-21	A
554	A	I	0	A
557	B	A	-1047	A
560	A	I	0	A
567	E	A	-40	A
571	B	A	-29	A
581	E	A	-74	A
583	A	I	0	A
599	E	A	-162	A
599	A	I	0	A
607	E	A	-69	A
609	A	I	0	A
610	B	A	-4	A
621	B	A	-115	A
722	A	I	0	A
729	B	A	-253	A
738	E	A	-608	A
740	B	A	-873	A
742	A	I	0	A
751	E	A	-2014	A
758	B	A	-234	A
762	B	A	-256	A
762	E	A	-527	A
762	A	I	0	A
772	B	A	-5422	A
774	E	A	-751	A
782	A	I	0	A
794	E	A	-1103	A

800	A	I	0	A
804	B	A	-1460	A
807	E	A	-5414	A
820	A	I	0	A
822	B	A	-4279	A
825	A	I	0	A
838	E	A	-650	A
850	A	I	0	A
869	E	A	-1127	A
871	B	A	-839	A
889	A	I	0	A
890	E	A	-953	A
890	B	A	-99	A
903	E	A	-53	A
906	B	A	-561	A
907	A	I	0	A
908	E	A	-7185	A
921	A	I	0	A
932	B	A	-239	A
933	A	I	0	A
934	E	A	-134	A
945	B	A	-6734	A
946	E	A	-9350	A
949	B	A	-323	A
959	A	I	0	A
984	E	A	-380	A
995	B	A	-861	A
996	A	I	0	A
1010	A	I	0	A
1027	E	A	-597	A
1036	B	A	-1	A
1036	E	A	-840	A
1046	A	I	0	A
1046	B	A	-30	A
1050	E	A	-248	A
1052	A	I	0	A
1055	B	A	-3	A
1066	B	A	-28	A
1066	E	A	-616	A
1072	B	A	-150	A
1076	E	A	-218	A
1079	A	I	0	A
1089	A	I	0	A
1122	E	A	-3009	A
1147	B	A	-39	A
1155	A	I	0	A
1192	B	A	-35	A

1194	E	A	-2212	A
1203	E	A	-824	A
1210	B	A	-201	A
1219	A	I	0	A
1222	A	I	0	A
1225	E	A	-1722	A
1240	A	I	0	A
1250	B	A	-73	A
1523	E	A	-152	A
1634	B	A	-283	A
1635	A	I	0	A
2797	E	A	-13154	A
2870	A	I	0	A
2872	B	A	-1613	A
3676	A	I	0	A
3676	B	A	-812	A

Table S19. Calculated IR and Raman active vibrations for $Bi(NH_4)[B(SO_4)_2]_4$ model 1.

Wavenumber / cm^{-1}	Irrep.	IR	Intensity / $km \cdot mol^{-1}$	Raman
0	B	A	0	A
0	E	A	0	A
56	E	A	5	A
64	E	A	24	A
66	B	A	3	A
69	E	A	130	A
75	B	A	112	A
79	E	A	10	A
84	B	A	155	A
87	E	A	85	A
88	B	A	113	A
89	B	A	116	A
90	E	A	31	A
93	B	A	2	A
97	E	A	412	A
98	B	A	56	A
104	E	A	297	A
105	B	A	8	A
107	B	A	59	A
110	E	A	92	A
112	B	A	183	A
120	E	A	249	A
124	E	A	25	A
125	B	A	17	A
132	B	A	130	A
137	E	A	79	A

141	B	A	2	A
152	E	A	46	A
153	B	A	0	A
158	E	A	49	A
162	B	A	106	A
164	E	A	97	A
178	E	A	56	A
187	E	A	44	A
189	B	A	2	A
209	E	A	4	A
210	B	A	3	A
215	E	A	6	A
217	E	A	8	A
217	B	A	2	A
220	B	A	1	A
235	B	A	132	A
239	E	A	7	A
246	B	A	76	A
247	E	A	41	A
251	B	A	0	A
255	E	A	4	A
259	E	A	48	A
269	E	A	16	A
273	B	A	40	A
278	E	A	143	A
278	B	A	16	A
283	B	A	123	A
311	E	A	1	A
322	B	A	1	A
326	E	A	162	A
338	B	A	251	A
341	E	A	4	A
358	B	A	65	A
359	E	A	17	A
360	E	A	135	A
366	B	A	45	A
367	B	A	169	A
380	B	A	3	A
381	E	A	35	A
384	E	A	370	A
401	E	A	147	A
410	E	A	2	A
413	B	A	15	A
421	B	A	20	A
426	B	A	927	A
438	E	A	48	A
441	E	A	231	A

447	B	A	1902	A
449	E	A	61	A
454	B	A	367	A
460	E	A	473	A
465	B	A	14	A
468	E	A	243	A
469	E	A	474	A
477	B	A	774	A
487	B	A	19	A
500	E	A	14	A
513	B	A	1	A
514	E	A	511	A
521	B	A	74	A
534	E	A	99	A
539	B	A	0	A
544	E	A	4725	A
546	B	A	4	A
562	B	A	1246	A
571	B	A	48	A
571	E	A	4	A
582	E	A	10	A
605	E	A	119	A
608	E	A	62	A
617	B	A	21	A
625	B	A	149	A
736	B	A	431	A
736	E	A	202	A
755	E	A	18	A
761	B	A	3155	A
763	B	A	1813	A
766	B	A	1241	A
787	E	A	188	A
788	E	A	646	A
804	E	A	8115	A
804	B	A	480	A
825	B	A	6111	A
841	E	A	225	A
874	E	A	189	A
877	B	A	284	A
885	E	A	365	A
887	B	A	9	A
895	E	A	1421	A
899	E	A	7028	A
900	B	A	210	A
927	B	A	89	A
929	E	A	700	A
942	B	A	6203	A

946	E	A	10123	A
951	B	A	1352	A
976	E	A	1	A
992	B	A	744	A
1018	E	A	166	A
1026	B	A	92	A
1032	E	A	906	A
1038	B	A	100	A
1042	E	A	3	A
1046	B	A	34	A
1056	E	A	624	A
1056	B	A	63	A
1064	B	A	1	A
1064	E	A	373	A
1181	B	A	0	A
1187	E	A	1665	A
1197	E	A	230	A
1198	B	A	232	A
1204	E	A	4822	A
1210	E	A	606	A
1211	B	A	369	A
1246	B	A	0	A
1431	E	A	497	A
1448	E	A	349	A
1449	B	A	409	A
1672	B	A	9	A
1684	B	A	3	A
3200	B	A	27	A
3309	E	A	2157	A
3331	B	A	1347	A
3331	E	A	1290	A

Wavenumber / cm^{-1}	Irrep.	IR	Intensity / km/mol	Raman
0	B	A	0	A
0	B	A	0	A
0	A	A	0	A
66	B	A	-54	A
71	A	A	-2	A
72	B	A	-34	A
74	A	A	-88	A
82	A	A	-38	A
85	B	A	-15	A
89	B	A	-54	A
89	A	A	-114	A
91	A	A	-6	A

96	A	A	-14	A
96	B	A	-139	A
101	A	A	-32	A
101	B	A	-29	A
105	B	A	-180	A
108	A	A	-19	A
119	B	A	-76	A
122	A	A	-113	A
125	A	A	-36	A
127	B	A	-35	A
134	A	A	-3	A
135	B	A	-26	A
139	B	A	-14	A
143	A	A	-1	A
155	A	A	-11	A
157	B	A	-12	A
162	B	A	-4	A
163	A	A	-53	A
166	B	A	-36	A
181	B	A	-2	A
187	B	A	-118	A
198	A	A	0	A
209	A	A	-2	A
214	B	A	-4	A
217	B	A	-11	A
233	A	A	-61	A
237	B	A	-2	A
242	A	A	-22	A
246	B	A	-12	A
246	A	A	-22	A
247	A	A	-17	A
251	B	A	-14	A
257	B	A	-12	A
259	A	A	0	A
261	B	A	-14	A
266	A	A	0	A
270	A	A	-11	A
277	B	A	-44	A
278	B	A	-22	A
281	A	A	-80	A
293	A	A	-1	A
322	B	A	-8	A
326	B	A	-63	A
328	B	A	-29	A
338	A	A	-142	A
358	A	A	-27	A
359	B	A	-39	A

361	B	A	-39	A
365	A	A	0	A
368	A	A	-112	A
382	B	A	-80	A
386	B	A	-87	A
388	A	A	-5	A
398	B	A	-36	A
407	B	A	-43	A
407	A	A	-1	A
421	A	A	-4	A
426	A	A	-517	A
437	A	A	0	A
438	B	A	-63	A
440	A	A	0	A
443	B	A	-93	A
448	A	A	-1164	A
459	B	A	-163	A
461	B	A	-63	A
465	B	A	-265	A
466	A	A	0	A
471	B	A	-152	A
477	A	A	-356	A
497	A	A	-21	A
510	A	A	-1	A
512	B	A	-181	A
516	B	A	-93	A
523	A	A	-38	A
540	A	A	0	A
543	B	A	-1166	A
543	B	A	-1235	A
560	A	A	-625	A
564	A	A	-24	A
580	B	A	-6	A
583	B	A	-5	A
602	B	A	-68	A
608	B	A	-23	A
610	A	A	0	A
625	A	A	-102	A
731	B	A	-84	A
740	B	A	-20	A
750	A	A	-913	A
761	A	A	-1567	A
764	A	A	-980	A
780	B	A	-244	A
790	A	A	-175	A
793	B	A	-893	A
799	B	A	-1029	A

814	B	A	-2357	A
817	A	A	-1239	A
826	A	A	-1789	A
867	A	A	-16	A
873	A	A	-73	A
883	B	A	-121	A
894	B	A	-3	A
897	B	A	-2317	A
901	A	A	-108	A
901	B	A	-2336	A
904	A	A	-61	A
924	A	A	-64	A
941	B	A	-2329	A
944	A	A	-2694	A
947	B	A	-2625	A
958	A	A	-924	A
995	A	A	-405	A
1015	B	A	-34	A
1025	B	A	-116	A
1032	B	A	-281	A
1034	B	A	-314	A
1039	A	A	-51	A
1046	A	A	0	A
1059	A	A	-59	A
1059	B	A	-403	A
1064	B	A	-141	A
1086	A	A	-4	A
1184	A	A	-1	A
1191	B	A	-742	A
1201	B	A	-57	A
1204	B	A	-1673	A
1210	A	A	-255	A
1210	B	A	-1208	A
1218	A	A	-29	A
1237	A	A	0	A
1436	B	A	-198	A
1444	B	A	-211	A
1451	A	A	-201	A
1672	A	A	-3	A
1686	A	A	-5	A
3209	A	A	-60	A
3304	B	A	-1110	A
3345	A	A	-631	A
3353	B	A	-616	A

References

[1] a) R. Hübenthal, *MAPLE. Programm for Calculation of the Madelung Part of Lattice Energy*, Universität Gießen, Gießen, **1993**; b) R. Hoppe, *Angew. Chem. Int. Ed.* **1966**, *5*, 95.

[2] a) T. Balić Žunić, E. Makovicky, *Acta Cryst.* **1996**, 78; b) E. Makovicky, T. Balić Žunić, *Acta Cryst.* **1998**, 766.
